# Novel approach to studying effects of inhalational exposure on lung function in civilians exposed to the World Trade Center disaster

**DOI:** 10.1038/s41598-023-30030-2

**Published:** 2023-02-24

**Authors:** Yuyan Wang, Kenneth I. Berger, Yian Zhang, Yongzhao Shao, Roberta M. Goldring, Joan Reibman, Mengling Liu

**Affiliations:** 1 Department of Population Health, New York University Grossman School of Medicine, 180 Madison Avenue, New York, NY 10016 USA; 2 Department of Medicine, New York University Grossman School of Medicine, 550 1st Avenue, New York, NY 10016 USA; 3 Department of Environmental Medicine, New York University Grossman School of Medicine, 550 1st Avenue, New York, NY 10016 USA

**Keywords:** Environmental impact, Risk factors, Respiratory tract diseases

## Abstract

It is increasingly important to study the impact of environmental inhalation exposures on human health in natural or man-made disasters in civilian populations. The members of the World Trade Center Environmental Health Center (WTC EHC; WTC Survivors) had complex exposures to environmental disaster from the destruction of WTC towers and can serve to reveal the effects of WTC exposure on the entire spectrum of lung functions. We aimed to investigate the associations between complex WTC exposures and measures of spirometry and oscillometry in WTC Survivors and included 3605 patients enrolled between Oct 1, 2009 and Mar 31, 2018. We performed latent class analysis and identified five latent exposure groups. We applied linear and quantile regressions to estimate the exposure effects on the means and various quantiles of pre-bronchodilator (BD) % predicted forced expiratory volume in one second (FEV_1_), forced vital capacity (FVC) and FEV_1_/FVC ratio, as well as the resistance at an oscillating frequency of 5 Hz (R_5_), frequency dependence of resistance R_5–20_, and reactance area (AX). Compared with Group 5, which had low or unknown exposure and was treated as the reference group, Group 1, the local workers with both acute and chronic exposures, had a lower median of % predicted FVC (−3.6; 95% CI: −5.4, −1.7) and higher (more abnormal) measures of AX at 10th quantile (0.77 cmH_2_O L^–1^ s; 95% CI: 0.41, 1.13) and 25th quantile (0.80 cmH_2_O L^−1^ s; 95% CI: 0.41, 1.20). Results suggested heterogeneous exposures to the WTC disaster had differential effects on the distributions of lung functions in the WTC Survivors. These findings could provide insights for future investigation of environmental disaster exposures.

## Introduction

Natural disasters such as large forest fires, desert dust storms, and man-made disasters continue to expose increasing numbers of civilians and responders to potentially toxic inhalations.^1–3^ These disasters usually involve complicated exposures, including the potential for short-term acute and/or long-term chronic multicomponent insults to the pulmonary system. In addition, the exposed populations are often diverse in age distribution, sex, race/ethnicity, socioeconomic status (SES), and baseline physical conditions including underlying lung function. The ability to assess the impact of complex exposures on large and small airways in a diverse population would allow for an improved understanding of inhalational injury.

The destruction of the World Trade Center (WTC) towers on September 11, 2001 was a massive environmental disaster, with the release of > 10 × 10^6^ tons of large and small particles in the surrounding area, with the potential for acute and chronic inhalation exposure to community members as well as responders.^1^ We and others have shown respiratory effects on large and small airways associated with the inhalation of this dust in civilian and responder populations.^4–8^ The World Trade Center Environmental Health Center (WTC EHC) is a “Center of Excellence” in the WTC Health Program (WTCHP), designed for members of the community (“Survivors”) with health symptoms due to their exposure to the WTC disaster in New York on 9/11/2001.^6,9,10^ In contrast to the WTCHP Centers of Excellence for Responders, members of the Survivor program are enrolled based on exposure as a local worker, resident, student, or present on 9/11 as a passerby, and presence of defined mental or physical health symptoms (https://www.nychealthandhospitals.org/services/wtc-environmental-health-center/). As a result of this, these Survivors have a much larger population diversity compared with WTC Responder cohorts.^11^ Survivors experienced complicated health hazards from the WTC disaster, including acute exposure to dust derived from the initial collapse of WTC towers, and chronic exposure to continued burning or agitation of the debris and fumes over the ensuing months.^12^ In addition, the Survivor cohort is unique in the WTCHP because its standardized measurements include both screening spirometry and respiratory oscillometry. We and others have recently shown the importance of oscillometry for the evaluation of the small airways in environmental exposures through the measurement of both respiratory resistance and reactance, and its importance in asthma is increasingly recognized.^5,7,13–15^ As such, WTC EHC Survivors are a unique and diverse population to study the effects of complex environmental exposures on the lung function of large and small airways.

To date, the association of complex exposures to the WTC disaster with large and small airway function measures has not been well explored. First, environmental exposure assessments are often simplified, and most studies focus on one exposure at a time. Two studies have applied principal component analyses to consider the complexity of multidimensional WTC exposures and to create composite WTC exposure scales based on responses to detailed WTC exposure questions.^6,10^ However, studies using principal components analyses have to exclude participants with missing values for any variables and use the derived multiple component variables. In contrast, latent class analysis (LCA) is a useful method for exposure analysis with the advantage that it defines subgroups by considering multiple variables concurrently.^16,17^ LCA has the potential to define heterogeneous exposure patterns by relating observed acute and chronic exposures including missing information to a set of discrete latent variables to generate mutually exclusive groups, thus enhancing the analysis of complex exposures. Second, most disease assessments only analyze group means or medians, which could miss the effects of complex exposures on the mild or severe disease at the ends of the disease spectrum. The analysis of the association between exposures and extremes in the spectrum of lung function (e.g., those with severe lung function abnormalities or those with more normal lung function) may provide additional information about the potential for lung injury. In addition, some measures of lung function (e.g., oscillometry) usually have a skewed distribution, therefore, directly applying approaches such as linear regression when modeling assumptions are not met, may yield biased results.^4,10^ Quantile regression can be used to address these issues and to examine the associations by directly characterizing conditional quantiles of the outcome, including adjustment for potential confounders. Quantile regression can also be more robust to the outliers and avoid transformations, thus allowing for simple interpretation.^18,19^.

This study focused on WTC Survivors and aimed to investigate the heterogeneous exposure patterns of complex WTC exposures using latent class analysis and to estimate the associations of exposures along the entire spectrum of lung function measurements using quantile regression. We hypothesized that the combination of LCA and quantile regression would reveal the effects of WTC exposures on lung function that may not have been detected when using standard analyses. Moreover, since the presence of more severely abnormal lung function might overwhelm the effects of exposures, we suggested that this combined approach might provide insight into subtle effects in a general population and improve our understanding of the health impact of man-made as well as natural disasters.

## Methods

### Study design and population

The WTC EHC was launched in 2005 and is now a Center of Excellence in the WTCHP under the Centers for Disease Control and Prevention (CDC) and the National Institute of Occupational Health (NIOSH) under the James Zadroga 9/11 Health and Compensation Act. Enrollees undergo a standardized physical and mental health evaluation.^11^ By law, the patients enrolled in this program must have a WTC-related diagnosis (defined aerodigestive disorders, acute traumatic injury, cancer, post-traumatic stress, depression, anxiety), a requirement that differs from the WTC Responder programs and thus yields a cohort with all patients having diagnostic conditions. Only patients who provided written informed consent were included for analysis, and the Institutional Review Board of New York University School of Medicine approved the research database (NCT00404898). All procedures and methods in studies involving human participants were performed in accordance with the relevant guidelines, regulations, and ethical standards of the New York University School of Medicine and the 2013 Helsinki Declaration and its later amendments or comparable ethical standards.^20^.

### Procedures

At enrollment, patients respond to a multi-dimensional interviewer-administered questionnaire including basic demographic information, WTC-related and other like occupational exposures, potential exposure category as a local worker, resident, student, and passerby, detailed description of upper and lower respiratory symptoms, as well as gastroesophageal, neurologic and mental health symptoms. Patients also undergo standardized clinical evaluations, including pre- and post-bronchodilator (BD) spirometry and oscillometry at the Bellevue Hospital site.

This study focused on patients enrolled between Oct 1, 2009 and Mar 31, 2018 at the Bellevue Hospital site, due to updates of exposure questions in the questionnaire and availability of pulmonary measurements. Based on the responses about residence and workplace, patients were categorized into three groups as Local workers, Local residents, and Others. If the patients reported being in the WTC dust cloud from the collapsing buildings on 9/11, they were classified as caught in the WTC cloud. A total of 15 exposure variables were included in the analyses and summarized in Supplementary table 1. Baseline covariates included for multivariable analyses were: age at the initial interview, sex, race/ethnicity groups (non-Hispanic White, non-Hispanic Black, Hispanic, non-Hispanic Asian, and non-Hispanic Other), body mass index (BMI) category (Underweight/normal < 25 kg/m^2^, Overweight 25–30 kg/m^2^, and Obese > 30 kg/m^2^), income group (< 15 K, 15 K–30 K, and > 30 K), and ever smoking status (No/Yes).

### Lung function measurements

Spirometry was performed on a Viasys Vmax spirometer (Viasys Healthcare, Yorba Linda, CA) in accordance with American Thoracic Society/European Respiratory Society standards.^21^ Data were excluded due to low quality before they were sent to the database. Spirometry data were electronically downloaded along with an automated quality assurance code. All studies were performed in the Bellevue Hospital Center Pulmonary Function (PFT) Laboratory. Data for pre-BD forced expiratory volume in one second (FEV_1_), forced vital capacity (FVC), and FEV_1_/FVC were analyzed based on predictive equations from the National Health and Nutrition Examination Survey (NHANES) III. Larger values of these spirometry measures indicate better lung function.

Oscillometry (IOS) was measured using the forced oscillation technique with the Jaeger Impulse Oscillation System (Jaeger, USA) in accordance with published recommendations.^22,23^ Maneuvers were performed during tidal breathing in the seated position, with a nose clip in place and with support of the cheeks. A minimum of three trials, each lasting 30 s, were performed. The volume versus time tracings were analyzed and only data from trials with nearly constant tidal volume and end-expiratory volume were included. Reproducibility between trials (variability < 10%) was required, and average values were derived for each subject. Oscillometry measures considered in this analysis included pre-BD resistance measured at an oscillating frequency of 5 Hz (R_5_), pre-BD frequency dependence of resistance (FDR) calculated as the difference between R_5_ and R_20_ (R_5–20_), and pre-BD reactance area (AX) calculated as the area above the reactance curve from 5 Hz to resonant frequency (${f}_{\mathrm{res}}$).^22^ In contrast to spirometry measures, lower values of these oscillometry measures indicate better lung function.

### Statistical analysis

We applied latent class analysis (LCA) to the observed exposure variables and categorized patients into mutually exclusive exposure pattern groups differentiated by values of unobserved latent variables. The number of latent classes was determined based on both model fit and latent class explanation. Outliers (outside 3 times of interquartile for the first or third quartile) of the lung function measures were excluded from the further analyses. We presented the summary statistics for patient characteristics, including counts and proportions for categorical variables; mean and standard deviation (SD) or median and interquartile (IQR) for continuous variables; and various quantiles for spirometry or oscillometry measures. Comparisons across the exposure groups were conducted using the analysis of variance (ANOVA) test or Kruskal–Wallis test for continuous variables and the Chi-square test for categorical variables. Multivariable linear regressions were conducted to estimate the difference in means of spirometry and log-transformed oscillometry measures across different exposure groups. To estimate the effects of WTC-exposures on the full spectrum of lung function measures, we applied quantile regressions considering spirometry and oscillometry measures as dependent variables to estimate the differences in conditional quantiles (10th, 25th, 50th, 75th, and 90th percentiles) between exposure groups, and modeling on log-transformed measures to estimate relative effect (%) on each quantile level, adjusting for baseline covariates.^24^ In addition, we conducted sensitivity analyses stratified by sex and BMI category and compared the coefficients among stratified groups. A *p*-value less than 0.001 was considered statistical significance to account for multiple testing, and all tests were two-sided. All statistical analyses were performed in R (version 4.0.0).

## Results

### Included patients

**Figure 1:**
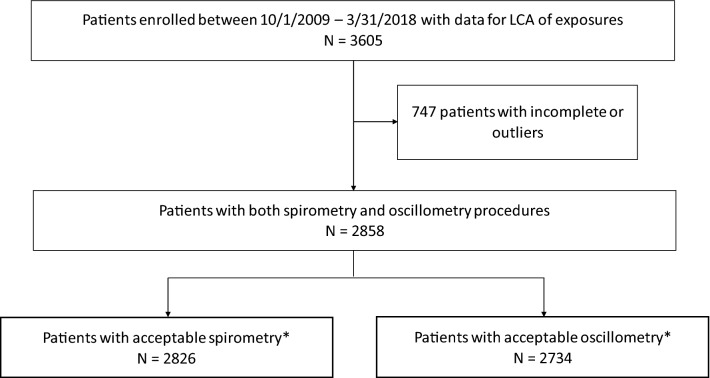
Patient flow diagram. ‘Acceptable’ means that the patients had all spirometry/oscillometry measures used in the analyses. LCA: latent class analysis.

Figure 1 shows the flowchart of the patients included in the analyses. There were 3605 patients enrolled in the WTC EHC at Bellevue Hospital who signed research consent between Oct 1, 2009 and Mar 31, 2018 and completed the initial questionnaire. These patients were used for the LCA analysis. For lung function analyses, we excluded patients who did not have complete spirometry or IOS data, resulting in 2826 patients with spirometry data and 2734 patients with IOS data, respectively.

### Latent class analysis of WTC exposures

**Figure 2:**
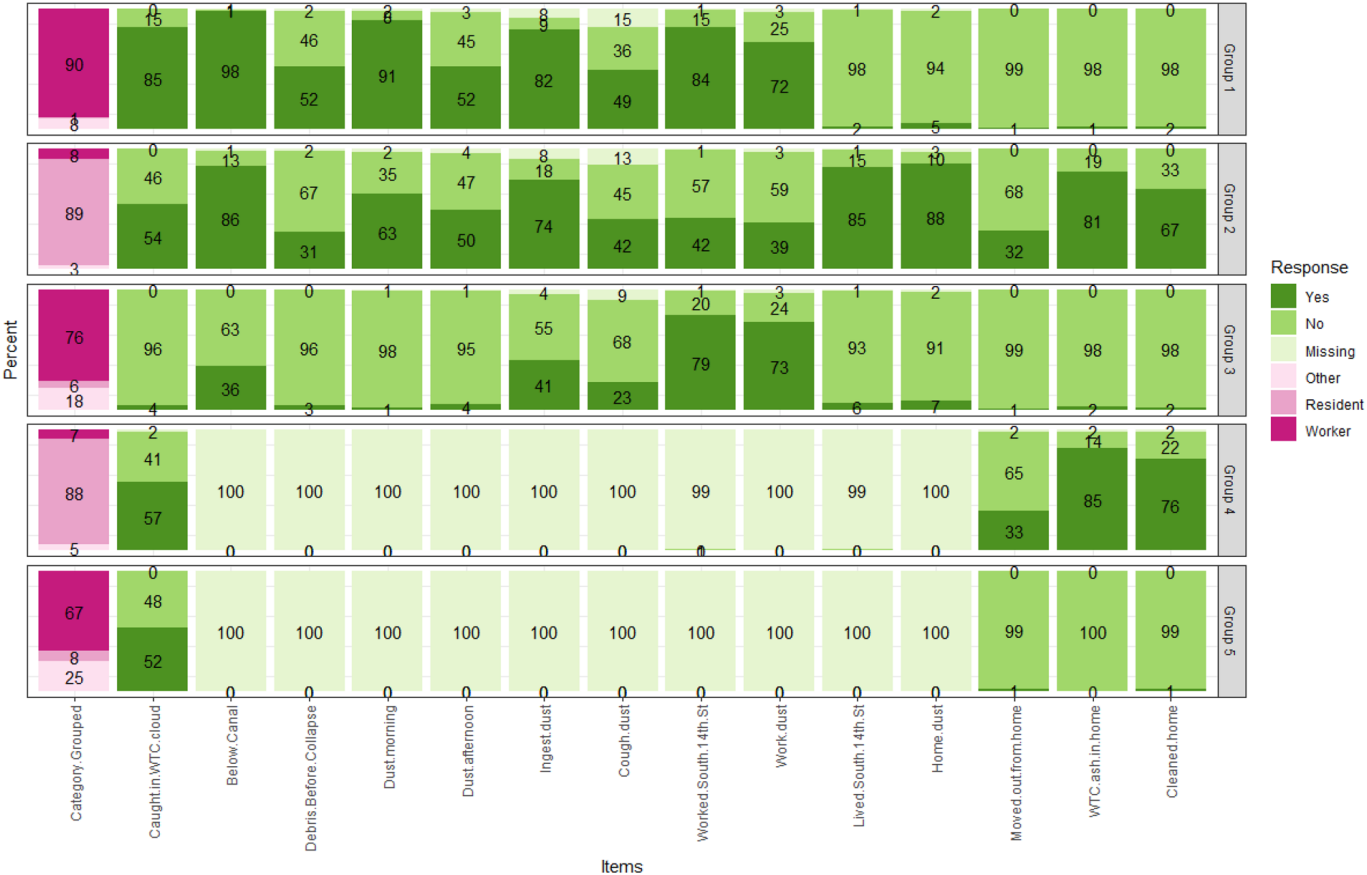
Latent class analysis with five latent exposure groups. X-axis lists 15 exposure variables used in latent class analysis, and y-axis represents the percent of exposure categories in each latent group. The analysis was based on 3605 patients. Five latent exposure groups were identified: 1348 (37.4%) local workers with acute and chronic exposures (Group 1); 789 (21.9%) local residents with acute and chronic exposures (Group 2); 516 (14.3%) local workers with chronic exposures (Group 3); 247 (6.9%) local residents with unknown acute exposures but high home exposure risks (Group 4); and 705 (19.6%) mixed patients with unknown acute exposures and low home exposure risks (Group 5).

To characterize the complex WTC exposures, we performed LCA based on the 15 acute and chronic exposure variables (Supplementary table 1). Figure 2 shows the five exposure groups identified by the LCA analysis. We interpreted these five groups as:

- Group 1 (n = 1348; 37.4%) included predominantly local workers with acute exposures on 9/11 (e.g., caught in the WTC dust cloud and ingesting dust) and chronic exposures (e.g., dust at the workplace).
- Group 2 (n = 789; 21.9%) consisted of predominantly residents with both acute and chronic exposures (e.g., ash/dust in the home).
- Group 3 (n = 516, 14.3%) included predominantly local workers with a high proportion of reported chronic work-related exposures (e.g., worked south of 14th Street; workplace with visible dust), but few reports of acute exposure.
- Group 4 (n = 247; 6.9%) included predominantly residents with unknown acute exposure but reports of chronic exposures in the home.
- Group 5 (n = 705; 19.6%) was a group of mixed local workers and others with some dust cloud exposure, but little information on other acute exposures, and minimal home exposures. Group 5 was used as the reference group in all subsequent analyses.

Both Groups 1 and 3 were mainly local workers and had similar work-related exposures. We considered that Group 1 had higher exposure than Group 3 because Group 1 had higher proportions of being caught in the dust cloud and covered by dust in the morning or afternoon. Similarly, Groups 2 and 4 were mainly local residents living in the area, but Group 2 reported much more acute exposures than Group 4. Therefore, Group 1 and Group 2 were considered as the most exposed, Group 3 and 4 followed with less exposure, and Group 5 was the least exposed or with unknown exposures.

### Patient characteristics

Characteristics of the 3605 patients overall and in five LCA exposure groups are summarized in Table 1. The WTC EHC Survivor population in this study was diverse with 50.0% female, 19.2% non-Hispanic Black, 18.6% Hispanic, and 12.4% non-Hispanic Asian. The majority of the population was overweight (28.8%) or obese (28.6%), with a broad distribution of individual income, and the ever-smoking rate was 39.5%. These patient characteristics were distributed differently among the five groups except for ever-smoking (Table 1). Specifically, Groups 1, 2, and 3 were older than Groups 4 and 5; the residents in Groups 2 and 4 included more females than males; Group 2 had a higher proportion of non-Hispanic Asians; and higher exposed Groups 1 and 2 had higher incomes compared with other Groups.

**Table 1:** Characteristics of patients in five latent exposure groups.

	Overall	Group 1	Group 2	Group 3	Group 4	Group 5	*p*-value
n	3605	1348	789	516	247	705	
Age at initial visit, y, mean ± SD	54.3 ± 12.9	55.7 ± 10.7	56.7 ± 15.1	55.1 ± 10.9	49.7 ± 17.1	49.9 ± 12.4	< 0.001
Age on 9/11/2001, y, mean ± SD	41.8 ± 12.5	42.0 ± 10.5	42.9 ± 14.7	41.4 ± 10.7	40.4 ± 17.2	40.8 ± 12.3	0.008
Sex, n (%)							0.007
Female	1802 (50.0)	633 (47.0)	427 (54.1)	253 (49.0)	138 (55.9)	351 (49.8)	
Male	1803 (50.0)	715 (53.0)	362 (45.9)	263 (51.0)	109 (44.1)	354 (50.2)	
Race/ethnicity, n (%)							< 0.001
Non-Hispanic White	1715 (47.6)	681 (50.5)	374 (47.4)	280 (54.3)	141 (57.1)	239 (33.9)	
Non-Hispanic Black	693 (19.2)	322 (23.9)	71 (9.0)	88 (17.1)	35 (14.2)	177 (25.1)	
Hispanic	670 (18.6)	233 (17.3)	93 (11.8)	93 (18.0)	29 (11.7)	222 (31.5)	
Non-Hispanic Asian	448 (12.4)	76 (5.6)	242 (30.7)	46 (8.9)	36 (14.6)	48 (6.8)	
Non-Hispanic Other	79 (2.2)	36 (2.7)	< 10	< 10	< 10	19 (2.7)	
BMI group, kg/m^2^, n (%)							< 0.001
Underweight/normal (< 25)	947 (26.3)	317 (23.5)	129 (25.0)	225 (28.5)	89 (36.0)	187 (26.5)	
Overweight (25–29)	1038 (28.8)	420 (31.2)	151 (29.3)	178 (22.6)	61 (24.7)	228 (32.3)	
Obese (> 30)	1032 (28.6)	434 (32.2)	162 (31.4)	120 (15.2)	56 (22.7)	260 (36.9)	
Missing	588 (16.3)	177 (13.1)	74 (14.3)	266 (33.7)	41 (16.6)	30 (4.3)	
Income, n (%)							< 0.001
≤ 15 K	1393 (38.6)	419 (31.1)	154 (29.8)	354 (44.9)	142 (57.5)	324 (46.0)	
15–30 K	468 (13.0)	165 (12.2)	54 (10.5)	120 (15.2)	33 (13.4)	96 (13.6)	
> 30 K	1744 (48.4)	764 (56.7)	308 (59.7)	315 (39.9)	72 (29.1)	285 (40.4)	
Smoking, n (%)							0.177
No	2180 (60.5)	841 (62.4)	307 (59.5)	485 (61.5)	140 (56.7)	407 (57.7)	
Yes	1425 (39.5)	507 (37.6)	209 (40.5)	304 (38.5)	107 (43.3)	298 (42.3)	

We first examined mean spirometry and median oscillometry values overall and in the five LCA exposure groups (Table 2). Mean spirometry of % predicted FEV_1_ and FVC were in the normal range for all groups; Group 1 had the lowest means (87.6% predicted FEV_1_ and 90.7% predicted FVC). Group 1 also had the most elevated median R_5–20_ (1.12 cmH_2_O L^–1^ s) and AX (8.19) compared with other groups. Measures of R_5–20_ and AX were significantly different across exposure groups. The means of FEV_1_/FVC and medians of R_5_ were similar across LCA exposure groups. Although the differences in spirometry means and oscillometry medians among the groups reached statistical significance, the magnitudes of differences for group means and medians were small.

**Table 2:** Summary of spirometry and oscillometry measures.

	Overall	Group 1	Group 2	Group 3	Group 4	Group 5	*p*-value
Spirometry measures, n	2826	1084	489	413	215	625	
% Predicted FEV_1_, mean ± SD	89.0 ± 17.9	87.6 ± 18.1	89.1 ± 17.6	88.2 ± 18.4	91.5 ± 17.4	91.0 ± 17.1	0.001
% Predicted FVC, mean ± SD	92.4 ± 16.3	90.7 ± 16.1	93.3 ± 16.1	91.4 ± 15.8	95.6 ± 16.4	93.9 ± 16.6	< 0.001
FEV_1_/FVC ratio, mean ± SD	75.6 ± 9.0	75.3 ± 9.2	74.6 ± 9.0	75.3 ± 9.9	75.8 ± 8.8	76.9 ± 8.0	< 0.001
Oscillometry measures, n	2734	1062	474	403	206	589	
R_5_, cmH_2_O L^–1^ s, median (IQR)	4.60 (3.59, 5.93)	4.66 (3.65, 6.03)	4.46 (3.49, 5.66)	4.49 (3.58, 5.66)	4.47 (3.42, 5.87)	4.68 (3.68, 6.16)	0.018
R_5–20_, cmH_2_O L^–1^ s, median (IQR)	1.03 (0.57, 1.74)	1.12 (0.66, 1.84)	0.94 (0.47, 1.59)	1.02 (0.59, 1.54)	0.94 (0.41, 1.69)	1.00 (0.55, 1.81)	< 0.001
AX, median (IQR)	6.91 (3.36, 14.28)	8.19 (3.90, 15.48)	5.88 (2.91, 12.85)	6.93 (3.54, 13.14)	6.12 (2.30, 13.09)	6.27 (3.01, 15.17)	< 0.001

### Lung function measures assessed using linear regression

We next applied multivariable linear regression with covariates adjustment to estimate the exposure effects on the average levels of lung function measures using Group 5 (with the least exposure) as reference. Because the distributions of oscillometry measures were right-skewed, we applied log-transformation first, and interpreted the results as the relative change (%) in the geometric mean of the outcome when the predictor increased one unit. Results are shown in Supplementary tables 2 and 3. The means of % predicted FEV_1_ and FVC were significantly reduced in Group 1 compared to Group 5 (% predicted FEV_1_, −3.1; 95% CI: −4.9, −1.3; % predicted FEV_1_, −2.9; 95% CI: −4.4, −1.3). Geometric means of R_5–20_ and AX, but not R_5_, were elevated in Group 1 compared to Group 5 (R_5–20_, 13.76%; 95% CI: 4.99, 23.27; AX, 20.44%; 95% CI: 9.71, 32.23). Age and obesity were associated with all measures, with the older and obese patients having worse lung function measures. Males had better oscillometry measures compared with females. Those who identified as the Black or Hispanic had worse oscillometry measures compared with the non-Hispanic White. Smoking was associated with a lower FEV_1_/FVC ratio.

### Lung function measures assessed using quantile regression

**Figure 3:**
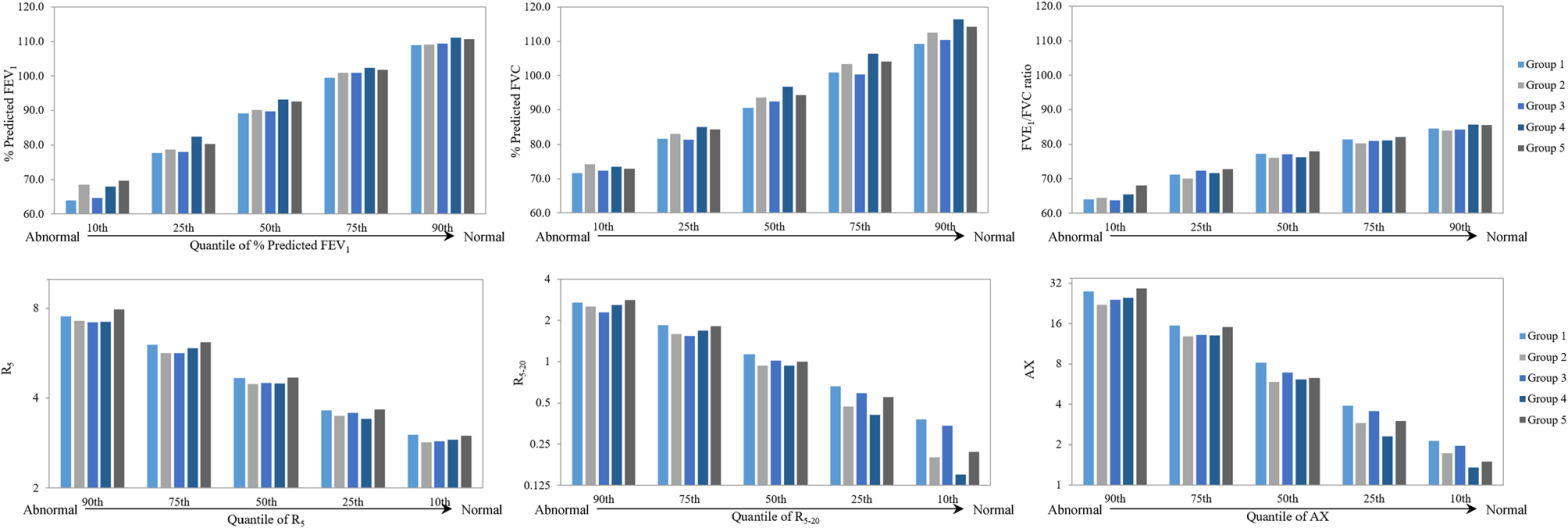
Quantiles of spirometry and oscillometry measures in five latent exposure groups. X-axis denotes five quantile levels, and y-axis is the quantile spirometry or oscillometry value within each group.

To evaluate whether WTC exposures were associated with lung function across different quantile levels, we created quantiles of spirometry and oscillometry measures in each LCA exposure group. Figure 3 shows the quantile values of spirometry and oscillometry measures within each LCA exposure group without adjustment for covariates, and the raw numbers are presented in Supplementary table 4. Compared with other groups, Group 1 (with the most acute and chronic exposure) had the lowest % predicted FEV_1_ and % predicted FVC at all quantile levels, and Group 1 also had the most elevated oscillometry values at most quantile levels, especially for the lower R_5–20_ and AX quantile (the more normal) levels.

**Figure 4:**
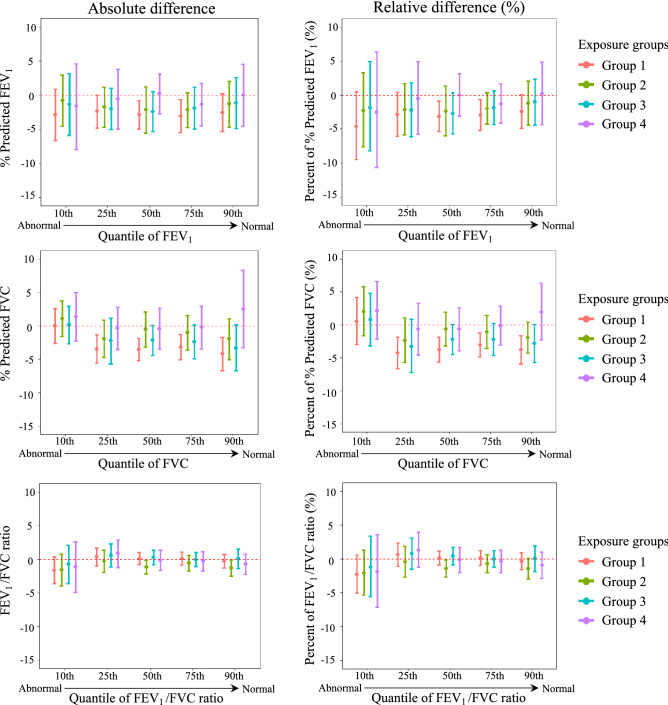
Estimates of absolute and relative effects on quantiles of % predicted FEV1, % predicted FVC, and FEV1/FVC ratio. X-axis denotes five quantile levels, and y-axis is the estimated absolute or relative difference on % predicted FEV1, % predicted FVC, and FEV1/FVC ratio. The colors represent four latent Group 1–4 vs. Group 5 (reference). The horizontal red dash line represents zero null effect. FEV1: pre- bronchodilator forced expiratory volume in one second. FVC: pre- bronchodilator forced vital capacity.

To further explore the associations between exposure and lung function across quantile levels, we used quantile regression to quantify the difference in lung function metrics after adjustment for covariates. Figure 4 shows the estimated absolute and relative difference in spirometry quantile values between each group and reference Group 5 (numbers in Supplementary tables 5 and 6). There were no significant differences at the 10th percentile (more abnormal level) in any group compared with Group 5. In contrast, % predicted FVC was lower in Group 1 compared with Group 5 from the 25th to 90th percentiles and reached a statistically significant difference at the median (50th percentile) level (absolute: −3. 6; 95%CI: −5.4, −1.7; relative: −3.8; 95% CI: −5.4, −2.0). No significant difference was noted for FEV_1_/FVC ratio at all quantile levels.

**Figure 5:**
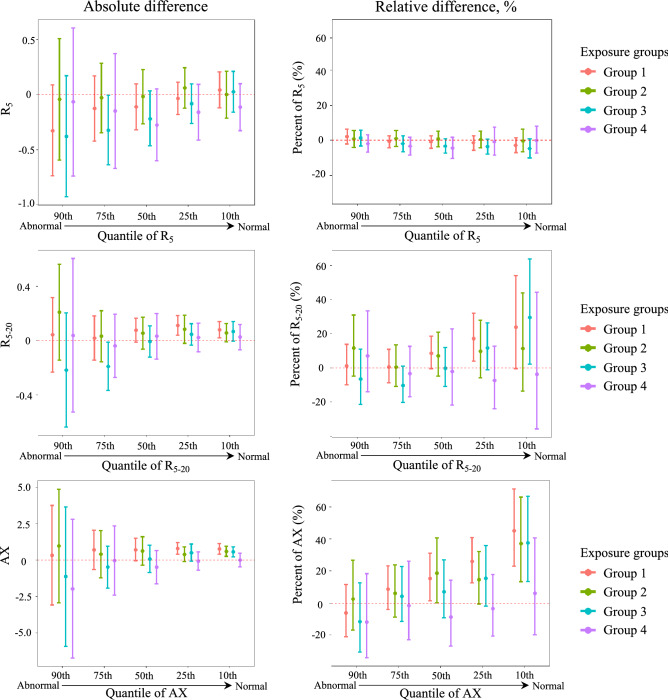
Estimates of absolute and relative effects on quantiles of R_5_, R_5–20_, and AX. X-axis denotes five quantile levels, and y-axis is the estimated absolute or relative difference on R_5_, R_5–20_, and AX. The colors represent four latent Group 1–4 vs. Group 5 (reference). The horizontal red dash line represents zero null effect. R_5_: pre-bronchodilator total airway resistance to 5 Hz. R_5–20_: pre-bronchodilator frequency dependence of resistance as difference between resistance at 5 Hz and 20 Hz. AX: pre-bronchodilator reactance curve area below zero.

To further examine small airway function, we applied the same quantile regression analysis for oscillometry measures. Figure 5 shows the estimates of covariates-adjusted absolute and relative differences in R_5_, R_5–20_, and AX between Groups 1 to 4 and reference Group 5, respectively (numbers in Supplementary tables 7 and 8). At the 90th percentile of oscillometry measures (more abnormal level), all groups were similar in all three oscillometry measures. In contrast, measures of small airway function including R_5–20_ and AX were more abnormal in Group 1 compared to Group 5 at 25th and 10th percentiles. There were significant differences of AX at 25th percentile (absolute: 0.80; 95% CI: 0.41, 1.20; relative: 25.92; 95% CI: 12.63, 40.77) and 10th percentile (absolute: 0.77; 95% CI: 0.41, 1.13; relative: 45.07; 95% CI: 22.83, 71.32). Evaluation of the effects highlighted that the relative differences in R_5–20_ and AX were 23.93% and 45.07% higher at the 10th percentile in Group 1 compared with Group 5. Notably, although the absolute differences were similar across the quantile levels, there was an obvious increase trend in AX relative differences by quantile levels for Group 1 compared with Group 5 (red bars in the right bottom plot of Fig. 5; from 90 to 10th percentile: −6.03%, 8.74%, 15.39%, 25.92%, 45.07%), suggesting that the effect of severe acute and chronic exposures on small airway function was most pronounced in patients with more normal lung function.

### Stratified sensitivity analyses

The sex-stratified sensitivity analyses using multivariable linear regressions and quantile regressions for spirometry and IOS measures did not show significant differences in the effects of exposures and covariates on lung function measures between males and females (Supplementary tables 9 and 10). We also conducted sensitivity analyses stratified by BMI category and no significant difference was found in the effects of exposure groups among BMI groups (Supplementary tables 11 and 12).

## Discussion

This study is based on data from the WTC EHC Survivor population: civilians with large population diversity with acute and/or chronic WTC disaster environmental exposure. We analyzed complex exposure patterns and their health impact across the spectrum of lung function measured by spirometry and oscillometry. Our results reinforced the need to evaluate the complexity of environmental exposures and the exposure impact across the full span of lung function, rather than only focusing on the mean or median of the population or subgroups. We showed that the complex WTC disaster exposure can be classified as different exposure patterns, and the magnitude of influence from these exposures on lung function differed among the exposure groups and varied along the quantiles of lung function measures. Quantile regression results indicated that severe acute and chronic WTC disaster exposure was more strongly associated with reduced lung function in the healthier spectrums of small airway parameters, i.e., lower AX, in the exposed populations. The greater effect of complex exposures at the more normal quantiles of lung function suggests that our study may identify early changes in lung injury and unmask findings that would not otherwise have been detected. These findings, and the applications of innovative statistical methods for inhalational analysis, are not only important for WTC exposures but also have implications for studies of other environmental exposures.

The attack on the WTC towers on 9/11 resulted in the pulverization and collapse of the two main structures and adjacent buildings, and fires that burned through December 2001 with the development of a large plume of dust and smoke that released both particles and gases into the atmosphere.^1^ The toxic components released by the destruction of the WTC towers were difficult to characterize due to the absence of samples and the chemical transformations by high-temperature combustion.^11^ Exposure patterns to the dust and fumes were also difficult to characterize due to differences in location and duration in the affected area. Local community members were at high risk from both acute exposures to the debris and dust clouds created by the collapsing buildings, as well as chronic exposures to airborne fumes and dust derived from continued burning or agitation of the debris.^11^ Because of the complexity of exposure in the civilian population, the impacts of different WTC exposures on this population are under-studied.

The diversity of individuals enrolled in the WTC EHC Survivor population and the variability in their exposure to the WTC dust/fumes may enhance our understanding of the impact of this environmental disaster on human health. A valuable understanding of the dose–response relationship to the WTC dust/fumes has been derived from studies of the Fire Department of NY (FDNY); a population that is predominantly male and White. In contrast, the WTC Survivors, who worked or lived in the local community around Ground Zero, are very diverse in sex, race/ethnicity, socioeconomic class and education and thus may be more representative of a civilian population exposed to a disaster. For example, 50% of WTC EHC survivors are female, and nearly half self-reported as Black, Hispanic, Asian, or other.

Many community members in or around the site were not only heavily immersed in scattered debris and massive clouds of pulverized dust, but also chronically exposed to re-suspended indoor and outdoor settled dust after returning to their workplaces and homes. Settled dust included a mixture of building debris and combustion products (mass median aerodynamic diameter > 23 μm), a blended mixture of concrete, gypsum, and synthetic vitreous fibers, with metals, radionuclides, ionic species, and asbestos.^1,12,25^ However, only a few studies have assessed the acute and chronic WTC disaster-related exposures on community members.^6,10^ We explored the complex WTC exposures using latent class analysis (LCA) by specifying different numbers of classes prior to the application. To choose different class groupings, we compared the LCA information criteria and domain-usefulness and included both the diversity among groups and the simplicity of interpretation.^26^ Our results showed that LCA created five latent groups based on multidimensional acute and chronic exposure information, representing the heterogeneity of exposure patterns in the WTC EHC Survivors, and also demonstrated that the LCA approach could be a useful tool to simplify complicated exposures. For example, Group 1 patients were mostly local workers caught in the WTC cloud (acute exposure) and had chronic work exposure; few reported exposure from living in the area. In contrast, Group 3 were also local workers with similar chronic work exposure but reported much less acute exposure. These mutually exclusive exposure groups facilitate the interpretation of health effects by combining massive exposure information with objective functional outcomes to identify specific inhalational risks.

There was a large spread of spirometry and oscillometry values, suggesting the need for more complex analyses on the full spectrum of lung function. Quantile analysis showed that only changes in % predicted FVC were significantly associated with exposure groups at medium or higher FVC levels. This finding is consistent with results from previous studies showing that while most WTC EHC patients have normal spirometry, the most common spirometry abnormality was a low FVC.^27^ The previous longitudinal analysis from our group also showed that whereas spirometry improved in many, it failed to return to the normal range in the low FVC or low FVC/obstructed groups.^28^ Moreover, we found that the persistence of abnormal FVC was compatible with persistent small airway dysfunction.^29^ These studies reinforced a potential mechanism of lung injury in this population, which might result from air trapping causing a “pseudorestrictive” pattern^29^, and our current results are consistent with these previous findings.

Oscillometry was performed routinely in this cohort as a potential measure of small airway involvement with the theory that oscillometry measures could detect airway injury earlier than conventional spirometry.^5,22,30^ Research has shown that oscillometry may be more sensitive than spirometry at identifying pathology in the peripheral airways.^31^ For example, AX represents reactance and provides information about the distensibility of airways (respiratory system dynamic elastance/distensibility and inertia) and can be viewed as complementary to frequency dependence of resistance.^32,33^ Our study uncovered significant associations between specific WTC exposures and small airway metrics that was evident in the lower spectrum (more normal) of AX and R_5–20_ distributions with decreased effect at the higher quantile levels. These results reaffirm that WTC exposures impact the peripheral lung, and more importantly extend prior observations by demonstrating an effect even in the healthier spectrum of small airway oscillometry metrics. We suggest that environmental exposure impacts the population in a diverse fashion with more detectable changes in the healthier spectrum of the distribution. Thus, traditional approaches that only focus on group mean values may not completely capture the effects of environmental exposures on lung function, particularly in those with more normal lung function. For individuals with higher FVC and lower AX and R_5–20_, the novel coupling of quantile regression with LCA uncovered potential exposure related inhalational lung injury located predominately in the lung periphery.

This study has several important strengths. The large sample size, population diversity, and exposure diversity in WTC EHC provide a unique opportunity to evaluate the influence of WTC disaster exposure on lung function. LCA is applied to summarize the complex exposure variables and to generate exclusively heterogeneous exposure patterns. To our knowledge, this study is the first to apply quantile regressions to assess the associations between WTC exposures and different quantiles of pulmonary function in WTC programs. Our study also has limitations. Our analysis was limited to records between Oct 1, 2009 and Mar 31, 2018 due to the update of exposure questions in the questionnaire and the availability of pulmonary measurements, but the relatively large sample size ensures the power to detect associations. The study is also subject to selection and recalls biases because this was a self-referred population whose enrollment depended on the presence of a defined diagnosis and the potential for exposure to WTC dust.^27^ Patients with missing spirometry or oscillometry measures were generally older, higher missing BMI, lower-income and were more likely to be Asian, which might explain to some extent the low compliance of conducting lung function measurements due to age and language barrier. Current analysis only used the lung function measured at the initial visit, and our future plans would include repeated measurements and focus on their influence on the longitudinal changes.^8,28,34–36^ This study did not consider asthma symptoms while previous studies from our group showed that some patients had persistent uncontrolled asthma symptoms despite treatment with inhaled corticosteroids.^37,38^ Not all confounders like post-traumatic stress disorder (PTSD) and anxiety that might influence pulmonary function were included in this study.^4,37,39^ This study did not consider occupational exposures or exposures from other natural or man-made disasters.

In conclusion, we analyzed the complex exposures to an environmental disaster using a group of exposure variables in a civilian population exposed to the WTC disaster and showed heterogeneity of exposure patterns with the identification of five mutually exclusive groups using LCA. The quantile regression results uncovered signs of airway injury, predominantly in small airways, that were within specific LCA exposure classes and surprisingly, were most evident in individuals with lung function at the normal range. These findings would not have been detected when using a single exposure or group means or medians. Our findings highlight the importance of using novel statistical methods that account for diversity in exposures and the whole distribution of lung function to uncover environmental injury in WTC as well as in other environmental exposures. This study also raises important issues about assessing the adverse health effects of future toxic inhalants and the potential to guide interventions to minimize risks to human health.

## Supplementary Information

Supplementary Information.

## Data Availability

The data that support the findings of this study are available from the WTC Health Program, but restrictions apply to the availability of these data, which were used under license for the current study, and so are not publicly available. Data are however available from the authors upon reasonable request and with permission of WTC Health Program. Dr. Reibman is the contact author for Data Availability details.
