# Supplementary material for: Novel approach to studying effects of inhalational exposure on lung function in civilians exposed to the World Trade Center disaster
Source: Sci Rep. 2023 Feb 24;13:3218. doi: 10.1038/s41598-023-30030-2 (PMC9958097; doi:10.1038/s41598-023-30030-2)
Supplement: Supplementary file 1 — Supplementary Information. [file 41598_2023_30030_MOESM1_ESM.docx]

**Supplemental Materials**

Novel approach to studying effects of inhalational exposure on lung function in civilians exposed to the World Trade Center disaster

Yuyan Wang^1^, Kenneth I. Berger^2^, Yian Zhang^1^, Yongzhao Shao^1,3^, Roberta M. Goldring^2^, Joan Reibman^2,3^, Mengling Liu^1,3^

^1^Department of Population Health, New York University Grossman School of Medicine, USA

^2^Department of Medicine, New York University Grossman School of Medicine, USA

^3^Department of Environmental Medicine, New York University Grossman School of Medicine, USA

**Correspondence to**:

Dr. Mengling Liu, New York University Grossman School of Medicine, 180 Madison Avenue, 554, New York, NY 10016, USA. E-mail: [mengling.liu@nyumc.org](mailto:mengling.liu@nyumc.org). Telephone: 646-501-3652.

Dr. Joan Reibman, New York University Grossman School of Medicine, 550 1st Avenue, Room NB7N24, New York, NY 10016, USA. E-mail: [joan.reibman@nyumc.org](mailto:joan.reibman@nyumc.org). Telephone: 212-263-6479.

**Supplementary table 1. Description and distribution of the 15 exposure variables used in latent class analysis**

|  | Exposure variables | Questions | Answers | | |
| --- | --- | --- | --- | --- | --- |
| 1 | Category.Grouped | WTC Categories: if a patient check both worker and resident, we will categorize him/her as local worker. | Local worker | Local resident | Other |
|  |  |  | 2164 (60.0) | 1021 (28.3) | 420 (11.7) |
|  |  |  | Yes | No | Missing |
| 2 | Caught.in.WTC.cloud | Were you caught in the WTC cloud of dust on September 11, 2001? | 2094 (58.1) | 1504 (41.7) | 7 (0.2) |
| 3 | Below.Canal | Were you below Canal Street on the day of September 11, 2001? | 2182 (60.5) | 451 (12.5) | 972 (27.0) |
| 4 | Debris.Before.Collapse | Were you caught in the WTC debris before the buildings collapsed on September 11, 2001? | 958 (26.6) | 1648 (45.7) | 999 (27.7) |
| 5 | Dust.morning | Were you caught in the WTC dust in the morning after the buildings collapsed on September 11, 2001? | 1723 (47.8) | 888 (24.6) | 994 (27.6) |
| 6 | Dust.afternoon | Were you caught in the WTC dust in the afternoon after the buildings collapsed on September 11, 2001? | 1108 (30.7) | 1472 (40.8) | 1025 (28.4) |
| 7 | Ingest.dust | Did you ingest, or swallow, any of the dust that came from the collapse of World Trade Center towers? | 1908 (52.9) | 555 (15.4) | 1142 (31.7) |
| 8 | Cough.dust | Did you cough up dust in the days after September 11, 2001? | 1102 (30.6) | 1192 (33.1) | 1311 (36.4) |
| 9 | Worked.South.14th.St | I worked in WTC area south of 14th street | 1879 (52.1) | 761 (21.1) | 965 (26.8) |
| 10 | Work.dust | In the year after September 11, 2001, did you work in an office, shop, restaurant, factory or other workplace in which WTC dust was visible on surfaces at any time, even if only briefly? | 1659 (46.0) | 925 (25.7) | 1021 (28.3) |
| 11 | Lived.South.14th.St | I lived (below) south of 14th street in Manhattan | 720 (20.0) | 1917 (53.2) | 968 (26.9) |
| 12 | Home.dust | In the year after September 11, 2001, did you live in an apartment or home in which WTC dust was visible on surfaces at any time, even if only briefly? | 793 (22.0) | 1809 (50.2) | 1003 (27.8) |
| 13 | Moved.out.from.home | Did you move out of your house immediately or soon after September 11, 2001 (within 2 weeks) because of the effects of September 11, 2001? | 366 (10.2) | 3232 (89.7) | 7 (0.2) |
| 14 | WTC.ash.in.home | Was the interior of your home covered in WTC dust? | 880 (24.4) | 2718 (75.4) | 7 (0.2) |
| 15 | Cleaned.home | Did you clean your own home of WTC dust? | 770 (21.4) | 2828 (78.4) | 7 (0.2) |

|  | Exposure variables | Group 1 (%) | | | Group 2 (%) | | | Group 3 (%) | | | Group 4 (%) | | | Group 5 (%) | | |
| --- | --- | --- | --- | --- | --- | --- | --- | --- | --- | --- | --- | --- | --- | --- | --- | --- |
|  |  | Local worker | Local resident | Other | Local worker | Local resident | Other | Local worker | Local resident | Other | Local worker | Local resident | Other | Local worker | Local resident | Other |
| 1 | Category.Grouped | 90.3 | 1.2 | 8.4 | 8.0 | 88.9 | 3.2 | 76.4 | 5.8 | 17.8 | 7.4 | 87.7 | 4.8 | 67.0 | 7.8 | 25.2 |
|  |  | Yes | No | Missing | Yes | No | Missing | Yes | No | Missing | Yes | No | Missing | Yes | No | Missing |
| 2 | Caught.in.WTC.cloud | 84.6 | 15.2 | 0.2 | 54.1 | 45.9 | 0.0 | 3.9 | 96.1 | 0.0 | 57.1 | 41.3 | 1.6 | 52.3 | 47.7 | 0.0 |
| 3 | Below.Canal | 98.0 | 1.5 | 0.6 | 85.6 | 12.9 | 1.5 | 36.4 | 63.3 | 0.3 | 0.0 | 0.0 | 100.0 | 0.1 | 0.0 | 99.9 |
| 4 | Debris.Before.Collapse | 51.7 | 46.0 | 2.3 | 31.1 | 67.0 | 1.9 | 3.5 | 96.2 | 0.3 | 0.0 | 0.0 | 100.0 | 0.0 | 0.0 | 100.0 |
| 5 | Dust.morning | 90.8 | 7.6 | 1.6 | 62.8 | 35.0 | 2.3 | 1.4 | 97.9 | 0.7 | 0.0 | 0.0 | 100.0 | 0.1 | 0.0 | 99.9 |
| 6 | Dust.afternoon | 51.6 | 45.4 | 3.1 | 49.7 | 46.7 | 3.6 | 4.5 | 94.9 | 0.7 | 0.0 | 0.0 | 100.0 | 0.0 | 0.0 | 100.0 |
| 7 | Ingest.dust | 82.5 | 9.5 | 8.1 | 74.1 | 18.0 | 7.8 | 41.3 | 54.9 | 3.8 | 0.0 | 0.0 | 100.0 | 0.0 | 0.0 | 100.0 |
| 8 | Cough.dust | 48.5 | 36.0 | 15.5 | 41.8 | 44.8 | 13.4 | 23.1 | 68.3 | 8.6 | 0.0 | 0.0 | 100.0 | 0.0 | 0.0 | 100.0 |
| 9 | Worked.South.14th.St | 84.3 | 15.1 | 0.6 | 42.5 | 57.0 | 0.5 | 78.9 | 20.5 | 0.7 | 0.0 | 0.8 | 99.2 | 0.0 | 0.0 | 100.0 |
| 10 | Work.dust | 72.3 | 25.0 | 2.7 | 38.7 | 58.8 | 2.5 | 73.2 | 24.1 | 2.8 | 0.0 | 0.0 | 100.0 | 0.1 | 0.0 | 99.9 |
| 11 | Lived.South.14th.St | 1.6 | 97.8 | 0.6 | 84.7 | 14.7 | 0.6 | 5.7 | 93.4 | 0.9 | 0.4 | 0.4 | 99.2 | 0.0 | 0.0 | 100.0 |
| 12 | Home.dust | 4.7 | 93.6 | 1.7 | 87.7 | 9.8 | 2.5 | 7.4 | 91.0 | 1.6 | 0.0 | 0.0 | 100.0 | 0.0 | 0.0 | 100.0 |
| 13 | Moved.out.from.home | 1.0 | 98.7 | 0.2 | 32.2 | 67.8 | 0.0 | 1.1 | 99.0 | 0.0 | 33.4 | 65.0 | 1.6 | 1.4 | 98.6 | 0.0 |
| 14 | WTC.ash.in.home | 1.4 | 98.4 | 0.2 | 80.8 | 19.2 | 0.0 | 2.3 | 97.7 | 0.0 | 84.8 | 13.6 | 1.6 | 0.1 | 100.0 | 0.0 |
| 15 | Cleaned.home | 2.2 | 97.6 | 0.2 | 67.2 | 32.8 | 0.0 | 2.2 | 97.8 | 0.0 | 76.2 | 22.2 | 1.6 | 1.2 | 98.8 | 0.0 |

**Supplementary table 2. Estimates of absolute effects on % predicted FEV_1_, % predicted FVC, and FEV_1_/FVC ratio from linear regressions**

|  | % Predicted FEV_1_ | P-value | % Predicted FVC | P-value | FEV_1_/FVC ratio | P-value |
| --- | --- | --- | --- | --- | --- | --- |
| Latent exposure group |  |  |  |  |  |  |
| Group 5 | Reference |  | Reference |  | Reference |  |
| Group 1 | -3.10 (-4.88, -1.31) | 0.00069 | -2.85 (-4.44, -1.26) | 0.00046 | -0.45 (-1.31, 0.41) | 0.30292 |
| Group 2 | -2.03 (-4.23, 0.16) | 0.06947 | -0.70 (-2.65, 1.26) | 0.48593 | -1.20 (-2.26, -0.14) | 0.02626 |
| Group 3 | -2.58 (-4.82, -0.33) | 0.02448 | -2.13 (-4.12, -0.13) | 0.03709 | -0.58 (-1.66, 0.50) | 0.29493 |
| Group 4 | -0.34 (-3.23, 2.56) | 0.81961 | 0.17 (-2.41, 2.74) | 0.89870 | -0.47 (-1.86, 0.92) | 0.51005 |
| Age at initial visit | -0.16 (-0.22, -0.10) | <0.00001 | -0.17 (-0.23, -0.12) | <0.00001 | -0.19 (-0.22, -0.16) | <0.00001 |
| Gender |  |  |  |  |  |  |
| Female | Reference |  | Reference |  | Reference |  |
| Male | -1.11 (-2.46, 0.24) | 0.10725 | -0.71 (-1.92, 0.49) | 0.24575 | -1.91 (-2.56, -1.26) | <0.00001 |
| Race/Ethnicity |  |  |  |  |  |  |
| White | Reference |  | Reference |  | Reference |  |
| Black | 0.86 (-0.94, 2.66) | 0.34808 | 1.04 (-0.57, 2.64) | 0.20517 | 1.52 (0.65, 2.38) | 0.00062 |
| Hispanic | -1.84 (-3.65, -0.03) | 0.04619 | -3.31 (-4.92, -1.70) | 0.00006 | 2.02 (1.15, 2.90) | 0.00001 |
| Asian | -5.00 (-7.66, -2.34) | 0.00023 | -9.11 (-11.48, -6.74) | <0.00001 | 3.53 (2.25, 4.81) | <0.00001 |
| Other | -9.70 (-14.28, -5.12) | 0.00003 | -10.82 (-14.90, -6.74) | <0.00001 | 1.09 (-1.11, 3.30) | 0.33081 |
| BMI group |  |  |  |  |  |  |
| Underweight/normal (<25) | Reference |  | Reference |  | Reference |  |
| Overweight (≥25 and <30) | -0.93 (-2.59, 0.74) | 0.27512 | -2.03 (-3.52, -0.55) | 0.00718 | 0.73 (-0.07, 1.53) | 0.07435 |
| Obese (≥30) | -5.18 (-6.88, -3.48) | <0.00001 | -7.29 (-8.81, -5.77) | <0.00001 | 1.34 (0.52, 2.16) | 0.00138 |
| Income group |  |  |  |  |  |  |
| <=15K | Reference |  | Reference |  | Reference |  |
| 15K-30K | -0.03 (-2.17, 2.10) | 0.97477 | 0.07 (-1.83, 1.97) | 0.94284 | -0.28 (-1.31, 0.74) | 0.58952 |
| >30K | 0.95 (-0.51, 2.40) | 0.20322 | 0.27 (-1.03, 1.57) | 0.68323 | 0.60 (-0.10, 1.30) | 0.09551 |
| Smoking |  |  |  |  |  |  |
| No | Reference |  | Reference |  | Reference |  |
| Yes | -2.16 (-3.52, -0.81) | 0.00180 | 0.47 (-0.74, 1.68) | 0.45049 | -2.25 (-2.90, -1.60) | <0.00001 |

**Supplementary table 3. Estimates of relative effects (%) on R_5_, R_5-20_, and AX from linear regressions**

|  | R_5_ | P-value | R_5-20_ | P-value | AX | P-value |
| --- | --- | --- | --- | --- | --- | --- |
| Latent exposure group |  |  |  |  |  |  |
| Group 5 | Reference |  | Reference |  | Reference |  |
| Group 1 | -1.42 (-4.63, 1.91) | 0.39870 | 13.76 (4.99, 23.27) | 0.00165 | 20.44 (9.71, 32.23) | 0.00010 |
| Group 2 | -0.55 (-4.52, 3.59) | 0.79074 | 11.88 (1.33, 23.53) | 0.02644 | 16.20 (3.61, 30.31) | 0.01033 |
| Group 3 | -3.32 (-7.25, 0.77) | 0.11038 | 8.52 (-1.87, 20.01) | 0.11123 | 11.35 (-0.92, 25.13) | 0.07123 |
| Group 4 | -4.07 (-9.07, 1.21) | 0.12882 | -1.52 (-13.59, 12.24) | 0.81877 | -3.41 (-16.92, 12.30) | 0.65159 |
| Age at initial visit | 0.25 (0.15, 0.36) | <0.00001 | 1.09 (0.83, 1.35) | <0.00001 | 1.53 (1.23, 1.83) | <0.00001 |
| Gender |  |  |  |  |  |  |
| Female | Reference |  | Reference |  | Reference |  |
| Male | -19.52 (-21.50, -17.48) | <0.00001 | -22.10 (-26.66, -17.26) | <0.00001 | -34.33 (-38.78, -29.56) | <0.00001 |
| Race/Ethnicity |  |  |  |  |  |  |
| White | Reference |  | Reference |  | Reference |  |
| Black | 7.93 (4.40, 11.59) | 0.00001 | 50.81 (39.21, 63.37) | <0.00001 | 71.49 (56.15, 88.33) | <0.00001 |
| Hispanic | 7.66 (4.14, 11.30) | 0.00001 | 32.00 (21.82, 43.02) | <0.00001 | 46.30 (33.24, 60.65) | <0.00001 |
| Asian | -0.55 (-5.35, 4.50) | 0.82836 | 8.92 (-3.49, 22.92) | 0.16623 | 11.90 (-2.64, 28.61) | 0.11345 |
| Other | 5.88 (-2.55, 15.05) | 0.17730 | 39.51 (14.22, 70.40) | 0.00112 | 43.60 (13.67, 81.40) | 0.00243 |
| BMI group |  |  |  |  |  |  |
| Underweight/normal (<25) | Reference |  | Reference |  | Reference |  |
| Overweight (≥25 and <30) | 13.98 (10.54, 17.53) | <0.00001 | 45.45 (35.00, 56.70) | <0.00001 | 39.95 (28.39, 52.56) | <0.00001 |
| Obese (≥30) | 35.55 (31.36, 39.87) | <0.00001 | 110.98 (95.55, 127.63) | <0.00001 | 126.51 (107.37, 147.42) | <0.00001 |
| Income group |  |  |  |  |  |  |
| <=15K | Reference |  | Reference |  | Reference |  |
| 15K-30K | 2.26 (-1.68, 6.37) | 0.26477 | 8.98 (-0.94, 19.89) | 0.07748 | 1.62 (-9.03, 13.51) | 0.77606 |
| >30K | -0.27 (-2.91, 2.45) | 0.84636 | -3.42 (-9.50, 3.07) | 0.29465 | -5.88 (-12.74, 1.51) | 0.11611 |
| Smoking |  |  |  |  |  |  |
| No | Reference |  | Reference |  | Reference |  |
| Yes | 1.44 (-1.07, 4.01) | 0.26449 | 2.26 (-3.75, 8.64) | 0.47022 | 9.56 (2.11, 17.56) | 0.01108 |

**Supplementary table 4. Quantile values of spirometry and oscillometry in each LCA exposure group (numbers for figure 3)**

| Quantile | 10th | 25th | 50th | 75th | 90th |
| --- | --- | --- | --- | --- | --- |
| % Predicted FEV_1_ |  |  |  |  |  |
| Group 1 | 64.0 | 77.7 | 89.1 | 99.5 | 109.0 |
| Group 2 | 68.5 | 78.7 | 90.2 | 100.9 | 109.1 |
| Group 3 | 64.6 | 78.0 | 89.8 | 101.0 | 109.4 |
| Group 4 | 67.9 | 82.4 | 93.2 | 102.4 | 111.1 |
| Group 5 | 69.7 | 80.3 | 92.6 | 101.8 | 110.7 |
| % Predicted FVC |  |  |  |  |  |
| Group 1 | 71.7 | 81.7 | 90.6 | 100.9 | 109.2 |
| Group 2 | 74.2 | 83.1 | 93.6 | 103.4 | 112.6 |
| Group 3 | 72.4 | 81.3 | 92.5 | 100.3 | 110.3 |
| Group 4 | 73.5 | 85.2 | 96.7 | 106.3 | 116.5 |
| Group 5 | 73.0 | 84.4 | 94.4 | 104.1 | 114.3 |
| FEV_1_/FVC ratio |  |  |  |  |  |
| Group 1 | 64.1 | 71.2 | 77.3 | 81.3 | 84.6 |
| Group 2 | 64.5 | 70.1 | 76.2 | 80.2 | 83.9 |
| Group 3 | 63.8 | 72.3 | 77.1 | 80.9 | 84.2 |
| Group 4 | 65.5 | 71.6 | 76.3 | 81.1 | 85.7 |
| Group 5 | 68.0 | 72.9 | 78.0 | 82.1 | 85.6 |
| R_5_, cmH_2_O·L^–1^·s |  |  |  |  |  |
| Group 1 | 3.02 | 3.65 | 4.66 | 6.03 | 7.52 |
| Group 2 | 2.85 | 3.49 | 4.46 | 5.66 | 7.26 |
| Group 3 | 2.87 | 3.58 | 4.49 | 5.66 | 7.18 |
| Group 4 | 2.91 | 3.42 | 4.47 | 5.87 | 7.20 |
| Group 5 | 3.00 | 3.68 | 4.68 | 6.16 | 7.94 |
| R_5-20_, cmH_2_O·L^–1^·s | |  |  |  |  |
| Group 1 | 0.38 | 0.66 | 1.13 | 1.84 | 2.71 |
| Group 2 | 0.20 | 0.47 | 0.94 | 1.59 | 2.54 |
| Group 3 | 0.34 | 0.59 | 1.02 | 1.54 | 2.29 |
| Group 4 | 0.15 | 0.41 | 0.94 | 1.69 | 2.59 |
| Group 5 | 0.22 | 0.55 | 1.00 | 1.81 | 2.82 |
| AX |  |  |  |  |  |
| Group 1 | 2.14 | 3.90 | 8.19 | 15.49 | 27.78 |
| Group 2 | 1.73 | 2.91 | 5.89 | 12.85 | 22.24 |
| Group 3 | 1.97 | 3.55 | 6.93 | 13.14 | 24.06 |
| Group 4 | 1.35 | 2.30 | 6.12 | 13.09 | 24.89 |
| Group 5 | 1.50 | 3.01 | 6.27 | 15.17 | 29.34 |

**Supplementary table 5. Estimates of absolute effects on % predicted FEV_1_, % predicted FVC, and FEV_1_/FVC ratio from quantile regressions (numbers for figure 4)**

|  | % Predicted FEV_1_ | P-value | % Predicted FVC | P-value | FEV_1_/FVC ratio | P-value |
| --- | --- | --- | --- | --- | --- | --- |
| Quantile=0.10 |  |  |  |  |  |  |
| Group 5 | Reference |  | Reference |  | Reference |  |
| Group 1 | -2.91 (-6.41, 0.59) | 0.10351 | -0.02 (-2.68, 2.65) | 0.99096 | -1.64 (-3.90, 0.62) | 0.15609 |
| Group 2 | -0.80 (-4.37, 2.78) | 0.66324 | 1.05 (-2.05, 4.16) | 0.50583 | -1.57 (-3.87, 0.73) | 0.18001 |
| Group 3 | -1.41 (-5.91, 3.10) | 0.53973 | 0.15 (-2.59, 2.89) | 0.91580 | -0.75 (-3.66, 2.16) | 0.61362 |
| Group 4 | -1.69 (-7.82, 4.43) | 0.58766 | 1.35 (-2.33, 5.03) | 0.47154 | -1.18 (-5.05, 2.69) | 0.54957 |
| Quantile=0.20 |  |  |  |  |  |  |
| Group 5 | Reference |  | Reference |  | Reference |  |
| Group 1 | -2.42 (-5.05, 0.20) | 0.07066 | -3.46 (-5.68, -1.23) | 0.00235 | 0.31 (-1.04, 1.66) | 0.65382 |
| Group 2 | -1.78 (-4.99, 1.44) | 0.2792 | -1.94 (-5.43, 1.55) | 0.27667 | -0.30 (-1.83, 1.22) | 0.69596 |
| Group 3 | -2.01 (-5.32, 1.31) | 0.23612 | -2.28 (-5.76, 1.21) | 0.20019 | 0.54 (-1.26, 2.34) | 0.55559 |
| Group 4 | -0.59 (-5.04, 3.87) | 0.79621 | -0.39 (-3.79, 3.01) | 0.82121 | 0.82 (-1.21, 2.85) | 0.42914 |
| Quantile=0.50 |  |  |  |  |  |  |
| Group 5 | Reference |  | Reference |  | Reference |  |
| Group 1 | -2.89 (-5.11, -0.66) | 0.01112 | -3.56 (-5.43, -1.68) | 0.00021 | 0.08 (-0.68, 0.84) | 0.83342 |
| Group 2 | -2.18 (-5.64, 1.27) | 0.21577 | -0.55 (-3.00, 1.90) | 0.65899 | -1.18 (-2.21, -0.15) | 0.02499 |
| Group 3 | -2.43 (-5.14, 0.28) | 0.07913 | -2.19 (-4.37, 0.00) | 0.04983 | 0.25 (-0.80, 1.31) | 0.63503 |
| Group 4 | 0.17 (-2.75, 3.08) | 0.90975 | -0.44 (-3.64, 2.77) | 0.78952 | -0.18 (-1.60, 1.23) | 0.79852 |
| Quantile=0.75 |  |  |  |  |  |  |
| Group 5 | Reference |  | Reference |  | Reference |  |
| Group 1 | -3.08 (-5.27, -0.89) | 0.00581 | -3.18 (-5.10, -1.26) | 0.00121 | 0.09 (-0.70, 0.88) | 0.82915 |
| Group 2 | -2.20 (-4.52, 0.12) | 0.06296 | -1.02 (-3.59, 1.55) | 0.43731 | -0.60 (-1.53, 0.32) | 0.20227 |
| Group 3 | -1.93 (-4.59, 0.74) | 0.15590 | -2.37 (-4.74, 0.00) | 0.04974 | -0.06 (-1.05, 0.93) | 0.90743 |
| Group 4 | -1.40 (-4.23, 1.42) | 0.32960 | -0.27 (-3.30, 2.76) | 0.86115 | -0.32 (-1.68, 1.04) | 0.6442 |
| Quantile=0.90 |  |  |  |  |  |  |
| Group 5 | Reference |  | Reference |  | Reference |  |
| Group 1 | -2.58 (-5.39, 0.23) | 0.07194 | -4.19 (-7.02, -1.35) | 0.00388 | -0.29 (-1.34, 0.75) | 0.58260 |
| Group 2 | -1.32 (-4.55, 1.92) | 0.42534 | -1.98 (-5.14, 1.19) | 0.22090 | -1.31 (-2.58, -0.05) | 0.04148 |
| Group 3 | -1.17 (-4.96, 2.62) | 0.54484 | -3.29 (-6.63, 0.04) | 0.05310 | 0.05 (-1.48, 1.58) | 0.94973 |
| Group 4 | -0.01 (-4.74, 4.72) | 0.99579 | 2.51 (-2.54, 7.56) | 0.33064 | -0.75 (-2.30, 0.80) | 0.34078 |

**Supplementary table 6. Estimates of relative effects (%) on % predicted FEV_1_, % predicted FVC, and FEV_1_/FVC ratio from quantile regressions (numbers for figure 4)**

|  | % Predicted FEV_1_ | P-value | % Predicted FVC | P-value | FEV_1/_FVC ratio | P-value |
| --- | --- | --- | --- | --- | --- | --- |
| Quantile=0.10 |  |  |  |  |  |  |
| Group 5 | Reference |  | Reference |  | Reference |  |
| Group 1 | -4.62 (-8.99, -0.04) | 0.04820 | 0.51 (-3.20, 4.36) | 0.79024 | -2.31 (-5.43, 0.91) | 0.15738 |
| Group 2 | -2.31 (-7.02, 2.65) | 0.35555 | 2.01 (-1.78, 5.94) | 0.30399 | -2.10 (-5.52, 1.45) | 0.24362 |
| Group 3 | -1.85 (-8.49, 5.27) | 0.60096 | 0.74 (-3.08, 4.71) | 0.70828 | -1.22 (-5.75, 3.52) | 0.60760 |
| Group 4 | -2.53 (-11.03, 6.78) | 0.58202 | 2.10 (-3.00, 7.47) | 0.42641 | -1.91 (-7.04, 3.49) | 0.48010 |
| Quantile=0.20 |  |  |  |  |  |  |
| Group 5 | Reference |  | Reference |  | Reference |  |
| Group 1 | -2.88 (-5.73, 0.06) | 0.05486 | -4.27 (-6.54, -1.94) | 0.00038 | 0.62 (-1.14, 2.42) | 0.49301 |
| Group 2 | -2.15 (-5.66, 1.50) | 0.24497 | -2.39 (-5.83, 1.18) | 0.18669 | -0.42 (-2.58, 1.79) | 0.70484 |
| Group 3 | -2.25 (-6.06, 1.71) | 0.26121 | -3.26 (-7.11, 0.76) | 0.11046 | 0.75 (-1.65, 3.20) | 0.54402 |
| Group 4 | -0.54 (-5.85, 5.06) | 0.84533 | -0.70 (-4.71, 3.49) | 0.74021 | 1.30 (-1.28, 3.96) | 0.32690 |
| Quantile=0.50 |  |  |  |  |  |  |
| Group 5 | Reference |  | Reference |  | Reference |  |
| Group 1 | -3.15 (-5.71, -0.52) | 0.01933 | -3.75 (-5.44, -2.04) | 0.00002 | 0.10 (-0.89, 1.10) | 0.84102 |
| Group 2 | -2.41 (-6.19, 1.53) | 0.22772 | -0.64 (-3.12, 1.91) | 0.62048 | -1.43 (-2.62, -0.22) | 0.02032 |
| Group 3 | -2.72 (-5.87, 0.54) | 0.10085 | -2.24 (-4.48, 0.04) | 0.05432 | 0.40 (-0.81, 1.62) | 0.52220 |
| Group 4 | -0.03 (-3.26, 3.30) | 0.98435 | -0.69 (-4.18, 2.93) | 0.70540 | -0.17 (-1.85, 1.54) | 0.84471 |
| Quantile=0.75 |  |  |  |  |  |  |
| Group 5 | Reference |  | Reference |  | Reference |  |
| Group 1 | -2.98 (-5.25, -0.66) | 0.01219 | -3.05 (-5.02, -1.05) | 0.00301 | 0.12 (-0.89, 1.14) | 0.82055 |
| Group 2 | -2.00 (-4.29, 0.34) | 0.09354 | -1.09 (-3.46, 1.34) | 0.37477 | -0.74 (-1.98, 0.52) | 0.25038 |
| Group 3 | -1.89 (-4.59, 0.89) | 0.18110 | -2.22 (-4.60, 0.22) | 0.07400 | -0.03 (-1.22, 1.17) | 0.95531 |
| Group 4 | -1.28 (-4.13, 1.66) | 0.38999 | -0.15 (-3.40, 3.20) | 0.92805 | -0.40 (-2.05, 1.28) | 0.63788 |
| Quantile=0.90 |  |  |  |  |  |  |
| Group 5 | Reference |  | Reference |  | Reference |  |
| Group 1 | -2.46 (-4.77, -0.10) | 0.04156 | -3.78 (-5.84, -1.68) | 0.00048 | -0.36 (-1.60, 0.89) | 0.56715 |
| Group 2 | -1.21 (-4.19, 1.86) | 0.43475 | -1.96 (-4.53, 0.68) | 0.14451 | -1.49 (-2.98, 0.02) | 0.05367 |
| Group 3 | -1.06 (-4.29, 2.28) | 0.52953 | -2.85 (-5.75, 0.13) | 0.06059 | 0.02 (-1.80, 1.87) | 0.98203 |
| Group 4 | 0.18 (-4.01, 4.55) | 0.93521 | 1.89 (-2.46, 6.44) | 0.39943 | -0.98 (-2.88, 0.97) | 0.32298 |

**Supplementary table 7. Estimates of absolute effects on R_5_, R_5-20_, and AX from quantile regressions (numbers for figure 5)**

|  | R_5_ | P-value | R_5-20_ | P-value | AX | P-value |
| --- | --- | --- | --- | --- | --- | --- |
| Quantile=0.10 |  |  |  |  |  |  |
| Group 5 | Reference |  | Reference |  | Reference |  |
| Group 1 | 0.04 (-0.12, 0.21) | 0.61582 | 0.08 (0.02, 0.14) | 0.01008 | 0.77 (0.41, 1.13) | 0.00003 |
| Group 2 | 0.00 (-0.21, 0.21) | 0.99373 | 0.06 (-0.01, 0.13) | 0.10593 | 0.60 (0.25, 0.94) | 0.00075 |
| Group 3 | 0.03 (-0.16, 0.21) | 0.78587 | 0.07 (-0.01, 0.14) | 0.07644 | 0.56 (0.22, 0.89) | 0.00103 |
| Group 4 | -0.11 (-0.33, 0.10) | 0.29930 | 0.03 (-0.07, 0.12) | 0.59338 | 0.00 (-0.48, 0.47) | 0.98559 |
| Quantile=0.20 |  |  |  |  |  |  |
| Group 5 | Reference |  | Reference |  | Reference |  |
| Group 1 | -0.03 (-0.18, 0.11) | 0.65081 | 0.11 (0.04, 0.18) | 0.00233 | 0.80 (0.41, 1.20) | 0.00007 |
| Group 2 | 0.06 (-0.12, 0.24) | 0.51360 | 0.08 (-0.02, 0.19) | 0.11056 | 0.40 (-0.11, 0.90) | 0.12220 |
| Group 3 | -0.08 (-0.26, 0.10) | 0.36882 | 0.05 (-0.03, 0.12) | 0.25945 | 0.51 (-0.07, 1.09) | 0.08639 |
| Group 4 | -0.16 (-0.41, 0.09) | 0.21720 | 0.02 (-0.08, 0.13) | 0.67425 | -0.07 (-0.72, 0.57) | 0.82463 |
| Quantile=0.50 |  |  |  |  |  |  |
| Group 5 | Reference |  | Reference |  | Reference |  |
| Group 1 | -0.11 (-0.32, 0.10) | 0.29704 | 0.08 (-0.01, 0.16) | 0.09167 | 0.71 (-0.07, 1.49) | 0.07388 |
| Group 2 | -0.02 (-0.26, 0.23) | 0.89030 | 0.05 (-0.06, 0.17) | 0.36154 | 0.62 (-0.37, 1.61) | 0.22002 |
| Group 3 | -0.22 (-0.46, 0.03) | 0.08842 | -0.01 (-0.12, 0.11) | 0.90022 | 0.08 (-0.86, 1.02) | 0.86373 |
| Group 4 | -0.27 (-0.60, 0.05) | 0.09973 | 0.03 (-0.13, 0.20) | 0.70556 | -0.49 (-1.63, 0.66) | 0.40623 |
| Quantile=0.75 |  |  |  |  |  |  |
| Group 5 | Reference |  | Reference |  | Reference |  |
| Group 1 | -0.13 (-0.42, 0.17) | 0.40767 | 0.02 (-0.14, 0.18) | 0.82797 | 0.71 (-0.65, 2.06) | 0.30590 |
| Group 2 | -0.03 (-0.34, 0.29) | 0.86235 | 0.03 (-0.16, 0.22) | 0.73827 | 0.40 (-1.23, 2.03) | 0.62967 |
| Group 3 | -0.32 (-0.64, -0.01) | 0.04612 | -0.19 (-0.37, -0.01) | 0.03517 | -0.48 (-1.91, 0.96) | 0.51482 |
| Group 4 | -0.15 (-0.67, 0.37) | 0.57683 | -0.04 (-0.27, 0.19) | 0.74842 | -0.03 (-2.41, 2.35) | 0.98025 |
| Quantile=0.90 |  |  |  |  |  |  |
| Group 5 | Reference |  | Reference |  | Reference |  |
| Group 1 | -0.32 (-0.74, 0.09) | 0.12352 | 0.04 (-0.23, 0.32) | 0.75989 | 0.34 (-3.09, 3.76) | 0.84732 |
| Group 2 | -0.04 (-0.59, 0.51) | 0.88385 | 0.21 (-0.14, 0.56) | 0.24732 | 0.98 (-2.93, 4.88) | 0.62432 |
| Group 3 | -0.38 (-0.93, 0.17) | 0.17816 | -0.22 (-0.64, 0.20) | 0.30981 | -1.13 (-5.92, 3.65) | 0.64253 |
| Group 4 | -0.07 (-0.74, 0.61) | 0.84911 | 0.04 (-0.53, 0.61) | 0.89464 | -1.96 (-6.72, 2.80) | 0.41996 |

**Supplementary table 8. Estimates of relative effects on R_5_, R_5-20_, and AX from quantile regressions (numbers for figure 5)**

|  | R_5_ | P-value | R_5-20_ | P-value | AX | P-value |
| --- | --- | --- | --- | --- | --- | --- |
| Quantile=0.10 |  |  |  |  |  |  |
| Group 5 | Reference |  | Reference |  | Reference |  |
| Group 1 | 2.22 (-2.97, 7.68) | 0.40864 | 23.93 (-0.40, 54.19) | 0.05444 | 45.07 (22.83, 71.32) | 0.00001 |
| Group 2 | 0.40 (-5.12, 6.24) | 0.88976 | 11.51 (-13.62, 43.94) | 0.40327 | 37.17 (13.31, 66.05) | 0.00120 |
| Group 3 | 1.09 (-4.60, 7.12) | 0.71369 | 29.52 (2.36, 63.91) | 0.03135 | 37.53 (13.46, 66.72) | 0.00119 |
| Group 4 | -2.81 (-9.60, 4.50) | 0.44102 | -3.65 (-35.68, 44.33) | 0.85696 | 6.17 (-19.81, 40.57) | 0.67589 |
| Quantile=0.20 |  |  |  |  |  |  |
| Group 5 | Reference |  | Reference |  | Reference |  |
| Group 1 | -1.35 (-5.54, 3.04) | 0.54112 | 17.18 (3.96, 32.07) | 0.00948 | 25.92 (12.63, 40.77) | 0.00005 |
| Group 2 | 0.90 (-4.34, 6.43) | 0.74070 | 9.81 (-5.72, 27.90) | 0.22910 | 14.64 (-0.53, 32.11) | 0.05928 |
| Group 3 | -2.86 (-8.06, 2.62) | 0.29994 | 11.77 (-1.07, 26.28) | 0.07401 | 15.46 (-1.85, 35.82) | 0.08293 |
| Group 4 | -4.63 (-10.79, 1.96) | 0.16420 | -7.29 (-23.83, 12.83) | 0.45000 | -3.38 (-20.71, 17.74) | 0.73348 |
| Quantile=0.50 |  |  |  |  |  |  |
| Group 5 | Reference |  | Reference |  | Reference |  |
| Group 1 | -1.55 (-5.74, 2.83) | 0.48254 | 8.65 (-0.35, 18.47) | 0.06004 | 15.39 (1.58, 31.09) | 0.02787 |
| Group 2 | 0.57 (-5.12, 6.60) | 0.84859 | 7.18 (-4.85, 20.74) | 0.25383 | 18.70 (0.18, 40.63) | 0.04766 |
| Group 3 | -4.49 (-9.61, 0.92) | 0.10227 | -0.05 (-10.88, 12.10) | 0.99355 | 7.23 (-9.28, 26.75) | 0.41305 |
| Group 4 | -5.95 (-12.70, 1.32) | 0.10658 | -1.94 (-21.68, 22.79) | 0.86470 | -8.65 (-26.96, 14.25) | 0.42810 |
| Quantile=0.75 |  |  |  |  |  |  |
| Group 5 | Reference |  | Reference |  | Reference |  |
| Group 1 | -2.30 (-7.97, 3.72) | 0.44627 | 0.64 (-8.78, 11.02) | 0.89914 | 8.74 (-3.91, 23.06) | 0.18432 |
| Group 2 | 0.19 (-5.78, 6.55) | 0.95119 | 0.53 (-10.93, 13.47) | 0.93143 | 6.27 (-8.78, 23.80) | 0.43516 |
| Group 3 | -4.90 (-10.43, 0.97) | 0.10008 | -10.19 (-20.28, 1.16) | 0.07693 | 4.33 (-11.30, 22.73) | 0.60856 |
| Group 4 | -1.42 (-10.02, 8.01) | 0.75940 | -3.20 (-16.82, 12.65) | 0.67412 | -1.45 (-22.97, 26.06) | 0.90721 |
| Quantile=0.90 |  |  |  |  |  |  |
| Group 5 | Reference |  | Reference |  | Reference |  |
| Group 1 | -3.85 (-9.08, 1.68) | 0.16866 | 1.27 (-9.84, 13.75) | 0.83086 | -6.03 (-20.89, 11.62) | 0.47900 |
| Group 2 | -0.73 (-8.42, 7.61) | 0.85923 | 11.72 (-4.67, 30.94) | 0.17114 | 2.67 (-16.75, 26.62) | 0.80561 |
| Group 3 | -6.19 (-12.78, 0.89) | 0.08544 | -6.51 (-21.36, 11.13) | 0.44522 | -11.56 (-30.64, 12.76) | 0.32181 |
| Group 4 | -0.35 (-8.42, 8.43) | 0.93481 | 7.15 (-13.94, 33.40) | 0.53705 | -11.76 (-34.16, 18.27) | 0.40265 |

**Supplementary table 9. Estimates of absolute effects on spirometry measures from linear regressions in male and female**

|  | % Predicted FEV_1_ | | |  | % Predicted FVC | | |  | FEV_1_/FVC ratio | | |
| --- | --- | --- | --- | --- | --- | --- | --- | --- | --- | --- | --- |
|  | Male | Female | P_value* |  | Male | Female | P-value* |  | Male | Female | P-value* |
| n | 1420 | 1406 |  |  | 1420 | 1406 |  |  | 1420 | 1406 |  |
| Exposure latent group |  |  |  |  |  |  |  |  |  |  |  |
| Group 5 | Reference | Reference |  |  | Reference | Reference |  |  | Reference | Reference |  |
|  |  |  |  |  |  |  |  |  |  |  |  |
| Group 1 | -2.54 (-5.08, 0.00) | -3.56 (-6.09, -1.03) | 0.57672 |  | -1.99 (-4.25, 0.27) | -3.58 (-5.84, -1.32) | 0.32927 |  | -0.91 (-2.21, 0.39) | -0.02 (-1.16, 1.12) | 0.31408 |
| Group 2 | -0.98 (-4.09, 2.14) | -2.97 (-6.07, 0.13) | 0.37398 |  | -0.05 (-2.82, 2.73) | -1.25 (-4.02, 1.51) | 0.54598 |  | -1.19 (-2.78, 0.41) | -1.18 (-2.58, 0.22) | 0.99614 |
| Group 3 | -2.07 (-5.22, 1.08) | -2.48 (-5.69, 0.74) | 0.85817 |  | -1.38 (-4.19, 1.42) | -2.42 (-5.29, 0.45) | 0.61305 |  | -1.09 (-2.70, 0.52) | 0.08 (-1.37, 1.53) | 0.29017 |
| Group 4 | -0.85 (-5.15, 3.45) | -0.20 (-4.12, 3.71) | 0.82768 |  | -0.27 (-4.11, 3.56) | 0.35 (-3.14, 3.85) | 0.81329 |  | -1.04 (-3.24, 1.16) | -0.07 (-1.83, 1.70) | 0.49789 |
| Age, yrs | -0.13 (-0.21, -0.05) | -0.19 (-0.27, -0.10) | 0.33221 |  | -0.16 (-0.23, -0.09) | -0.19 (-0.26, -0.11) | 0.66266 |  | -0.18 (-0.22, -0.14) | -0.20 (-0.24, -0.16) | 0.40281 |
| Race/Ethnicity |  |  |  |  |  |  |  |  |  |  |  |
| White | Reference | Reference |  |  | Reference | Reference |  |  | Reference | Reference |  |
| Black | -0.36 (-3.14, 2.42) | 0.84 (-1.63, 3.31) | 0.52599 |  | 0.41 (-2.07, 2.88) | 0.90 (-1.31, 3.10) | 0.77158 |  | 1.37 (-0.05, 2.80) | 1.43 (0.31, 2.54) | 0.95498 |
| Hispanic | -0.12 (-2.65, 2.41) | -3.80 (-6.46, -1.15) | 0.04928 |  | -1.41 (-3.67, 0.84) | -5.28 (-7.65, -2.91) | 0.02042 |  | 1.74 (0.44, 3.03) | 2.16 (0.97, 3.36) | 0.63558 |
| Asian | -4.28 (-8.14, -0.41) | -5.62 (-9.32, -1.91) | 0.62372 |  | -9.10 (-12.55, -5.65) | -9.10 (-12.40, -5.79) | 0.99972 |  | 4.01 (2.03, 5.99) | 3.12 (1.45, 4.79) | 0.49970 |
| Other | -8.76 (-15.47, -2.06) | -10.59 (-16.89, -4.28) | 0.69804 |  | -9.55 (-15.52, -3.58) | -12.10 (-17.73, -6.47) | 0.54293 |  | 0.64 (-2.79, 4.07) | 1.64 (-1.20, 4.49) | 0.65871 |
| BMI group |  |  |  |  |  |  |  |  |  |  |  |
| Underweight/normal (<25) | Reference | Reference |  |  | Reference | Reference |  |  | Reference | Reference |  |
| Overweight (≥25 and <30) | -2.06 (-4.45, 0.33) | -0.06 (-2.43, 2.30) | 0.24550 |  | -3.12 (-5.25, -0.98) | -1.05 (-3.16, 1.07) | 0.17627 |  | 0.81 (-0.42, 2.03) | 0.56 (-0.50, 1.63) | 0.77194 |
| Obese (≥30) | -7.08 (-9.60, -4.57) | -3.54 (-5.94, -1.14) | 0.04565 |  | -8.70 (-10.94, -6.46) | -6.09 (-8.23, -3.95) | 0.09854 |  | 1.02 (-0.27, 2.30) | 1.74 (0.66, 2.82) | 0.39851 |
| Income group |  |  |  |  |  |  |  |  |  |  |  |
| <=15K | Reference | Reference |  |  | Reference | Reference |  |  | Reference | Reference |  |
| 15K-30K | 2.39 (-0.61, 5.39) | -2.42 (-5.46, 0.62) | 0.02747 |  | 1.49 (-1.18, 4.17) | -1.37 (-4.08, 1.34) | 0.14066 |  | 0.51 (-1.02, 2.05) | -1.07 (-2.45, 0.30) | 0.13141 |
| >30K | 0.79 (-1.29, 2.86) | 1.09 (-0.95, 3.14) | 0.83671 |  | 0.48 (-1.37, 2.34) | 0.09 (-1.74, 1.92) | 0.76837 |  | 0.31 (-0.75, 1.38) | 0.85 (-0.07, 1.78) | 0.45392 |
| Smoking |  |  |  |  |  |  |  |  |  |  |  |
| No | Reference | Reference |  |  | Reference | Reference |  |  | Reference | Reference |  |
| Yes | -3.30 (-5.20, -1.41) | -1.08 (-3.04, 0.89) | 0.11009 |  | -0.20 (-1.89, 1.49) | 1.09 (-0.67, 2.84) | 0.30088 |  | -2.65 (-3.62, -1.68) | -1.86 (-2.74, -0.97) | 0.23830 |

* P-values are comparisons between the male and the female.

**Supplementary table 10. Estimates of absolute effects on oscillometry measures from quantile regressions in male and female**

|  | R_5_ | | |  | R_5_20_ | | |  | AX | | |
| --- | --- | --- | --- | --- | --- | --- | --- | --- | --- | --- | --- |
|  | Male | Female | P-value* |  | Male | Female | P-value* |  | Male | Female | P-value* |
| Quantile=0.10 |  |  |  |  |  |  |  |  |  |  |  |
| Group 5 | Reference | Reference |  |  | Reference | Reference |  |  | Reference | Reference |  |
| Group 1 | 0.05 (-0.14, 0.24) | 0.10 (-0.14, 0.35) | 0.75918 |  | 0.06 (-0.02, 0.14) | 0.13 (0.01, 0.24) | 0.37665 |  | 0.71 (0.28, 1.13) | 1.14 (0.46, 1.82) | 0.29482 |
| Group 2 | 0.07 (-0.16, 0.30) | -0.02 (-0.33, 0.28) | 0.61389 |  | 0.04 (-0.07, 0.15) | 0.08 (-0.03, 0.18) | 0.63054 |  | 0.63 (0.17, 1.09) | 0.64 (-0.06, 1.34) | 0.98136 |
| Group 3 | 0.01 (-0.25, 0.27) | 0.09 (-0.22, 0.40) | 0.7071 |  | 0.06 (-0.04, 0.16) | 0.12 (-0.01, 0.24) | 0.45939 |  | 0.46 (0.03, 0.89) | 0.89 (0.22, 1.56) | 0.28600 |
| Group 4 | -0.23 (-0.50, 0.04) | 0.14 (-0.16, 0.43) | 0.06951 |  | 0.01 (-0.13, 0.16) | 0.08 (-0.06, 0.23) | 0.5033 |  | 0.08 (-0.53, 0.69) | 0.32 (-0.36, 1.00) | 0.61657 |
| Quantile=0.20 |  |  |  |  |  |  |  |  |  |  |  |
| Group 5 | Reference | Reference |  |  | Reference | Reference |  |  | Reference | Reference |  |
| Group 1 | -0.08 (-0.31, 0.16) | -0.03 (-0.27, 0.20) | 0.80189 |  | 0.09 (-0.01, 0.20) | 0.05 (-0.08, 0.19) | 0.66268 |  | 0.22 (-0.29, 0.73) | 1.11 (0.24, 1.99) | 0.08700 |
| Group 2 | 0.08 (-0.18, 0.34) | 0.03 (-0.24, 0.29) | 0.79444 |  | 0.09 (-0.04, 0.23) | 0.01 (-0.13, 0.15) | 0.41513 |  | 0.32 (-0.38, 1.01) | 0.62 (-0.33, 1.57) | 0.61378 |
| Group 3 | -0.07 (-0.39, 0.26) | -0.14 (-0.43, 0.15) | 0.75576 |  | 0.02 (-0.10, 0.14) | 0.03 (-0.10, 0.15) | 0.96866 |  | 0.02 (-0.70, 0.73) | 0.98 (0.03, 1.94) | 0.11520 |
| Group 4 | -0.21 (-0.70, 0.27) | -0.09 (-0.38, 0.20) | 0.66854 |  | -0.03 (-0.19, 0.12) | 0.00 (-0.14, 0.15) | 0.75018 |  | -0.36 (-1.11, 0.39) | 0.57 (-0.52, 1.66) | 0.16988 |
| Quantile=0.50 |  |  |  |  |  |  |  |  |  |  |  |
| Group 5 | Reference | Reference |  |  | Reference | Reference |  |  | Reference | Reference |  |
| Group 1 | 0.00 (-0.23, 0.24) | -0.18 (-0.49, 0.12) | 0.34045 |  | 0.08 (-0.03, 0.19) | 0.12 (-0.02, 0.26) | 0.63464 |  | 0.51 (-0.40, 1.41) | 1.09 (-0.21, 2.39) | 0.47190 |
| Group 2 | 0.04 (-0.26, 0.34) | -0.22 (-0.60, 0.16) | 0.29744 |  | 0.11 (-0.03, 0.26) | 0.04 (-0.14, 0.22) | 0.53766 |  | 0.52 (-0.61, 1.64) | 0.99 (-0.44, 2.42) | 0.61118 |
| Group 3 | -0.13 (-0.44, 0.19) | -0.36 (-0.76, 0.04) | 0.37153 |  | 0.00 (-0.13, 0.14) | -0.01 (-0.17, 0.15) | 0.91073 |  | 0.09 (-1.00, 1.18) | 0.29 (-1.09, 1.67) | 0.82532 |
| Group 4 | -0.07 (-0.51, 0.38) | -0.40 (-0.92, 0.11) | 0.33127 |  | -0.03 (-0.29, 0.24) | 0.05 (-0.19, 0.28) | 0.68033 |  | -1.00 (-2.21, 0.21) | 0.30 (-1.24, 1.85) | 0.19492 |
| Quantile=0.75 |  |  |  |  |  |  |  |  |  |  |  |
| Group 5 | Reference | Reference |  |  | Reference | Reference |  |  | Reference | Reference |  |
| Group 1 | 0.14 (-0.30, 0.58) | -0.24 (-0.70, 0.21) | 0.24027 |  | 0.03 (-0.14, 0.20) | -0.11 (-0.40, 0.19) | 0.43900 |  | 0.92 (-0.62, 2.45) | -0.38 (-3.12, 2.36) | 0.42170 |
| Group 2 | 0.08 (-0.41, 0.57) | 0.09 (-0.46, 0.63) | 0.98221 |  | 0.05 (-0.16, 0.26) | -0.01 (-0.30, 0.28) | 0.75867 |  | 0.68 (-1.26, 2.62) | 0.08 (-3.05, 3.20) | 0.74945 |
| Group 3 | -0.22 (-0.67, 0.24) | -0.38 (-0.88, 0.13) | 0.64503 |  | -0.20 (-0.41, 0.01) | -0.33 (-0.61, -0.04) | 0.48953 |  | 0.26 (-1.64, 2.15) | -1.78 (-4.39, 0.82) | 0.21672 |
| Group 4 | 0.13 (-0.63, 0.90) | -0.23 (-0.93, 0.48) | 0.49876 |  | 0.02 (-0.40, 0.44) | -0.15 (-0.49, 0.19) | 0.53086 |  | 1.01 (-3.06, 5.08) | -0.66 (-4.18, 2.86) | 0.54540 |
| Quantile=0.90 |  |  |  |  |  |  |  |  |  |  |  |
| Group 5 | Reference | Reference |  |  | Reference | Reference |  |  | Reference | Reference |  |
| Group 1 | -0.02 (-0.63, 0.59) | -0.87 (-1.52, -0.23) | 0.05938 |  | 0.14 (-0.20, 0.49) | -0.21 (-0.60, 0.18) | 0.18735 |  | 2.03 (-2.57, 6.64) | -2.14 (-7.49, 3.22) | 0.25000 |
| Group 2 | 0.05 (-0.78, 0.88) | -0.42 (-1.38, 0.54) | 0.46959 |  | 0.30 (-0.19, 0.78) | 0.16 (-0.30, 0.63) | 0.70162 |  | 1.77 (-3.46, 7.01) | 1.92 (-5.13, 8.97) | 0.97405 |
| Group 3 | -0.13 (-0.92, 0.66) | -0.64 (-1.49, 0.20) | 0.38543 |  | -0.10 (-0.64, 0.44) | -0.04 (-0.54, 0.45) | 0.88227 |  | -0.30 (-7.47, 6.88) | -2.68 (-8.66, 3.30) | 0.61884 |
| Group 4 | 0.23 (-1.06, 1.52) | -0.46 (-1.39, 0.47) | 0.39597 |  | 0.68 (-0.05, 1.41) | -0.23 (-0.79, 0.32) | 0.05210 |  | 6.29 (-4.51, 17.08) | -4.22 (-9.93, 1.49) | 0.09363 |

* P-values are comparisons between the male and the female.

**Supplementary table 11. Estimates of absolute effects on spirometry measures from quantile regressions in BMI groups**

|  | % Predicted FEV_1_ | | |  | % Predicted FVC | | |  | FEV_1_/FVC Ratio | | |
| --- | --- | --- | --- | --- | --- | --- | --- | --- | --- | --- | --- |
|  | Normal | Overweight | Obese |  | Normal | Overweight | Obese |  | Normal | Overweight | Obese |
|  | n=846 | n=959 | n=942 |  | n=846 | n=959 | n=942 |  | n=846 | n=959 | n=942 |
| Group1 vs. Group0 |  |  |  |  |  |  |  |  |  |  |  |
| quantile=0.10 | -5.91 (-12.50, 0.67) | -0.72 (-7.58, 6.14) | -4.86 (-10.06, 0.34) |  | -2.52 (-8.10, 3.07) | 1.92 (-2.13, 5.97) | -0.01 (-4.08, 4.06) |  | -2.16 (-6.65, 2.32) | 1.48 (-1.54, 4.49) | -2.28 (-5.93, 1.37) |
| quantile=0.25 | -2.40 (-7.66, 2.87) | 0.19 (-3.69, 4.07) | -5.31 (-9.62, -0.99) |  | -4.78 (-8.61, -0.94) | -1.41 (-4.82, 2.00) | -4.51 (-8.39, -0.63) |  | 1.05 (-1.01, 3.10) | -0.36 (-2.55, 1.82) | -0.91 (-2.59, 0.76) |
| quantile=0.50 | -2.46 (-7.06, 2.15) | -1.54 (-5.33, 2.25) | -4.04 (-7.68, -0.41) |  | -3.31 (-6.88, 0.26) | -1.32 (-4.95, 2.30) | -4.23 (-7.12, -1.34) |  | 1.35 (-0.69, 3.39) | 0.25 (-1.14, 1.64) | -0.55 (-1.67, 0.58) |
| quantile=0.75 | -1.89 (-5.26, 1.49) | -3.48 (-7.72, 0.77) | -3.59 (-7.89, 0.71) |  | -3.49 (-7.48, 0.50) | -4.59 (-8.31, -0.87) | -2.12 (-5.04, 0.80) |  | 0.03 (-2.15, 2.21) | 0.32 (-0.83, 1.47) | -0.34 (-1.56, 0.87) |
| quantile=0.90 | -1.23 (-6.35, 3.89) | -2.45 (-7.18, 2.28) | -2.57 (-6.80, 1.65) |  | -4.01 (-8.60, 0.59) | -5.12 (-10.27, 0.03) | -4.73 (-9.93, 0.48) |  | -0.23 (-2.77, 2.30) | 0.01 (-1.45, 1.47) | -0.40 (-1.73, 0.94) |
| Group2 vs. Group0 |  |  |  |  |  |  |  |  |  |  |  |
| quantile=0.10 | -2.52 (-10.74, 5.71) | 0.19 (-8.09, 8.47) | -3.92 (-12.73, 4.89) |  | -0.11 (-6.77, 6.56) | -0.12 (-5.35, 5.11) | 0.90 (-3.69, 5.50) |  | -2.92 (-7.33, 1.50) | -0.92 (-6.93, 5.09) | -0.62 (-4.07, 2.83) |
| quantile=0.25 | -0.95 (-6.90, 5.00) | -1.77 (-6.97, 3.43) | -2.28 (-7.81, 3.25) |  | -2.92 (-6.94, 1.11) | 0.24 (-4.83, 5.30) | -3.80 (-8.64, 1.03) |  | 0.94 (-2.62, 4.50) | 0.55 (-2.41, 3.51) | -0.53 (-3.05, 1.99) |
| quantile=0.50 | -2.48 (-7.45, 2.50) | -0.32 (-5.86, 5.22) | -1.74 (-5.74, 2.25) |  | -2.12 (-6.39, 2.16) | 0.55 (-3.70, 4.80) | -3.38 (-6.82, 0.05) |  | 1.39 (-1.17, 3.96) | 0.76 (-1.21, 2.73) | -0.25 (-1.80, 1.29) |
| quantile=0.75 | -2.42 (-6.43, 1.60) | 0.99 (-3.30, 5.28) | -3.02 (-8.35, 2.31) |  | -4.40 (-8.92, 0.13) | -1.93 (-6.48, 2.62) | -2.42 (-5.99, 1.16) |  | -1.69 (-4.05, 0.67) | 0.89 (-0.36, 2.14) | -0.23 (-1.48, 1.02) |
| quantile=0.90 | -3.67 (-9.36, 2.01) | 1.75 (-3.57, 7.07) | -2.56 (-8.14, 3.02) |  | -6.30 (-12.19, -0.41) | -4.57 (-10.21, 1.08) | -4.68 (-12.07, 2.70) |  | -1.03 (-4.38, 2.32) | 0.76 (-1.27, 2.79) | -0.49 (-2.41, 1.44) |
| Group3 vs. Group0 |  |  |  |  |  |  |  |  |  |  |  |
| quantile=0.10 | -2.48 (-8.17, 3.21) | 0.46 (-7.67, 8.59) | -1.02 (-7.69, 5.64) |  | 0.46 (-5.48, 6.39) | 1.98 (-2.57, 6.54) | 1.06 (-5.46, 7.57) |  | -1.23 (-5.50, 3.03) | 1.39 (-2.53, 5.32) | -3.90 (-8.69, 0.89) |
| quantile=0.25 | -0.78 (-6.18, 4.62) | -0.91 (-6.77, 4.94) | -4.81 (-10.15, 0.53) |  | -3.01 (-7.28, 1.26) | -0.94 (-5.80, 3.92) | -4.19 (-10.08, 1.69) |  | 1.01 (-1.32, 3.35) | -2.36 (-4.83, 0.11) | -1.33 (-3.88, 1.22) |
| quantile=0.50 | -2.57 (-7.39, 2.25) | 0.11 (-4.54, 4.76) | -4.54 (-10.86, 1.78) |  | -0.81 (-4.60, 2.98) | 2.24 (-2.71, 7.19) | -1.93 (-6.14, 2.29) |  | 0.24 (-1.84, 2.31) | -2.13 (-3.91, -0.34) | -0.36 (-2.25, 1.52) |
| quantile=0.75 | -1.48 (-5.10, 2.14) | -0.33 (-4.51, 3.85) | -2.73 (-8.31, 2.84) |  | -2.81 (-6.96, 1.33) | -0.93 (-5.16, 3.30) | -1.73 (-6.88, 3.42) |  | -1.23 (-3.63, 1.17) | -0.26 (-2.04, 1.53) | -1.23 (-2.79, 0.34) |
| quantile=0.90 | -0.11 (-5.21, 4.99) | -0.94 (-5.93, 4.05) | -1.05 (-7.61, 5.50) |  | -2.09 (-6.35, 2.16) | -3.95 (-9.59, 1.69) | -2.39 (-10.47, 5.68) |  | -0.83 (-3.40, 1.74) | -0.91 (-2.95, 1.12) | -1.88 (-3.79, 0.03) |
| Group4 vs. Group0 |  |  |  |  |  |  |  |  |  |  |  |
| quantile=0.10 | 0.45 (-9.95, 10.85) | -1.03 (-11.07, 9.01) | -5.18 (-15.45, 5.09) |  | 0.11 (-8.74, 8.97) | 3.59 (-4.77, 11.96) | 2.99 (-4.04, 10.03) |  | 2.95 (-2.30, 8.21) | 4.19 (-1.99, 10.36) | -4.50 (-11.45, 2.45) |
| quantile=0.25 | 4.71 (-0.70, 10.13) | -2.08 (-9.40, 5.24) | -6.12 (-14.99, 2.75) |  | 0.07 (-5.90, 6.03) | 0.49 (-5.00, 5.98) | -3.31 (-10.12, 3.49) |  | 2.75 (0.32, 5.17) | -0.64 (-3.26, 1.99) | -3.55 (-9.99, 2.88) |
| quantile=0.50 | 1.67 (-3.36, 6.69) | -1.30 (-7.79, 5.20) | -0.60 (-8.03, 6.84) |  | 1.62 (-3.09, 6.34) | -1.08 (-6.73, 4.56) | -1.09 (-6.29, 4.11) |  | 1.08 (-1.51, 3.67) | -0.93 (-3.43, 1.56) | 0.68 (-2.01, 3.38) |
| quantile=0.75 | 0.76 (-4.01, 5.54) | -2.23 (-9.92, 5.46) | -3.35 (-8.28, 1.58) |  | -1.04 (-6.30, 4.22) | 1.40 (-9.16, 11.96) | -3.21 (-8.01, 1.60) |  | -1.14 (-3.42, 1.15) | 0.06 (-2.41, 2.53) | -0.05 (-2.24, 2.14) |
| quantile=0.90 | 5.63 (-3.24, 14.49) | 1.27 (-7.13, 9.66) | -7.83 (-14.67, -0.99) |  | 4.67 (-5.75, 15.08) | 1.78 (-6.51, 10.07) | -7.66 (-17.04, 1.71) |  | -1.56 (-4.67, 1.54) | -0.43 (-2.84, 1.98) | -0.48 (-2.87, 1.92) |

**Supplementary table 12. Estimates of absolute effects on oscillometry measures from quantile regressions in BMI groups**

|  | R_5_ | | |  | R_5_20_ | | |  | AX | | |
| --- | --- | --- | --- | --- | --- | --- | --- | --- | --- | --- | --- |
|  | Normal | Overweight | Obese |  | Normal | Overweight | Obese |  | Normal | Overweight | Obese |
|  | n=814 | n=933 | n=914 |  | n=814 | n=933 | n=914 |  | n=814 | n=933 | n=914 |
| Group1 vs. Group0 |  |  |  |  |  |  |  |  |  |  |  |
| quantile=0.10 | 0.17 (-0.04, 0.38) | 0.06 (-0.29, 0.40) | -0.10 (-0.47, 0.27) |  | 0.17 (0.05, 0.29) | 0.03 (-0.08, 0.15) | 0.02 (-0.15, 0.19) |  | 0.67 (0.15, 1.19) | 0.76 (0.26, 1.26) | 0.81 (-0.28, 1.91) |
| quantile=0.25 | 0.27 (0.03, 0.52) | -0.25 (-0.48, -0.02) | -0.16 (-0.45, 0.12) |  | 0.24 (0.13, 0.35) | 0.03 (-0.10, 0.16) | 0.08 (-0.11, 0.27) |  | 0.75 (0.13, 1.36) | 0.83 (0.22, 1.43) | 1.47 (-0.02, 2.97) |
| quantile=0.50 | 0.31 (0.01, 0.62) | -0.21 (-0.48, 0.07) | -0.30 (-0.76, 0.16) |  | 0.22 (0.10, 0.33) | 0.10 (-0.07, 0.27) | 0.04 (-0.13, 0.20) |  | 1.08 (0.27, 1.89) | 1.10 (0.03, 2.18) | 0.32 (-2.23, 2.87) |
| quantile=0.75 | 0.28 (-0.14, 0.70) | -0.12 (-0.60, 0.36) | -0.53 (-1.17, 0.12) |  | 0.23 (0.00, 0.47) | -0.08 (-0.31, 0.15) | 0.01 (-0.33, 0.35) |  | 1.25 (-0.46, 2.96) | 0.88 (-1.52, 3.28) | -1.50 (-5.70, 2.70) |
| quantile=0.90 | -0.03 (-0.86, 0.79) | -0.43 (-1.31, 0.44) | -0.48 (-1.18, 0.22) |  | 0.21 (-0.13, 0.55) | -0.08 (-0.60, 0.43) | -0.04 (-0.57, 0.49) |  | 2.93 (-2.82, 8.68) | 0.52 (-4.52, 5.56) | -1.66 (-11.21, 7.89) |
| Group2 vs. Group0 |  |  |  |  |  |  |  |  |  |  |  |
| quantile=0.10 | 0.12 (-0.16, 0.40) | -0.05 (-0.43, 0.33) | -0.07 (-0.51, 0.37) |  | 0.15 (0.01, 0.30) | 0.03 (-0.07, 0.14) | 0.04 (-0.20, 0.28) |  | 0.60 (0.07, 1.13) | 0.38 (-0.18, 0.94) | 1.35 (0.19, 2.52) |
| quantile=0.25 | 0.07 (-0.19, 0.33) | -0.33 (-0.70, 0.03) | -0.22 (-0.61, 0.17) |  | 0.16 (0.04, 0.29) | -0.04 (-0.19, 0.12) | 0.06 (-0.11, 0.23) |  | 0.41 (-0.38, 1.19) | 0.74 (-0.21, 1.69) | 1.27 (-0.25, 2.80) |
| quantile=0.50 | -0.02 (-0.48, 0.43) | -0.23 (-0.59, 0.14) | -0.31 (-0.85, 0.24) |  | 0.11 (-0.03, 0.26) | 0.05 (-0.12, 0.23) | -0.15 (-0.37, 0.08) |  | 0.40 (-0.59, 1.38) | 0.64 (-0.82, 2.09) | -0.27 (-3.01, 2.48) |
| quantile=0.75 | 0.06 (-0.45, 0.56) | -0.35 (-0.88, 0.19) | -0.79 (-1.45, -0.14) |  | 0.13 (-0.16, 0.43) | -0.22 (-0.46, 0.03) | -0.43 (-0.82, -0.04) |  | 0.87 (-1.29, 3.03) | 0.26 (-2.51, 3.03) | -2.76 (-7.87, 2.35) |
| quantile=0.90 | 0.00 (-1.17, 1.17) | -0.49 (-1.41, 0.44) | -0.39 (-1.36, 0.59) |  | 0.16 (-0.33, 0.65) | -0.40 (-1.00, 0.19) | -0.24 (-0.99, 0.51) |  | 2.94 (-7.76, 13.64) | -3.42 (-9.57, 2.74) | -5.46 (-15.43, 4.51) |
| Group3 vs. Group0 |  |  |  |  |  |  |  |  |  |  |  |
| quantile=0.10 | 0.24 (0.01, 0.47) | -0.20 (-0.59, 0.19) | -0.15 (-0.69, 0.38) |  | 0.12 (-0.01, 0.26) | -0.01 (-0.15, 0.13) | -0.02 (-0.24, 0.21) |  | 0.62 (0.03, 1.21) | 0.23 (-0.31, 0.76) | 0.97 (-0.37, 2.31) |
| quantile=0.25 | 0.38 (0.13, 0.63) | -0.24 (-0.54, 0.06) | 0.06 (-0.39, 0.50) |  | 0.12 (0.01, 0.23) | -0.01 (-0.15, 0.14) | 0.11 (-0.18, 0.41) |  | 0.57 (-0.05, 1.19) | 0.16 (-0.62, 0.93) | 1.42 (-0.69, 3.53) |
| quantile=0.50 | 0.34 (0.01, 0.66) | -0.15 (-0.59, 0.28) | -0.29 (-0.85, 0.26) |  | 0.15 (-0.01, 0.31) | 0.05 (-0.15, 0.25) | 0.14 (-0.15, 0.43) |  | 1.36 (0.43, 2.30) | 0.23 (-1.28, 1.75) | 0.92 (-2.13, 3.98) |
| quantile=0.75 | 0.36 (-0.10, 0.82) | 0.09 (-0.47, 0.65) | -0.54 (-1.25, 0.17) |  | 0.21 (-0.03, 0.45) | -0.03 (-0.33, 0.26) | 0.01 (-0.39, 0.41) |  | 0.79 (-1.01, 2.58) | 1.00 (-1.43, 3.42) | -1.25 (-7.19, 4.68) |
| quantile=0.90 | 0.28 (-0.59, 1.16) | -0.15 (-1.15, 0.85) | -0.52 (-1.84, 0.80) |  | 0.35 (-0.08, 0.78) | 0.05 (-0.61, 0.72) | 0.22 (-0.57, 1.02) |  | 4.17 (-1.39, 9.72) | 0.49 (-5.40, 6.38) | -2.18 (-12.53, 8.17) |
| Group4 vs. Group0 |  |  |  |  |  |  |  |  |  |  |  |
| quantile=0.10 | 0.19 (-0.05, 0.43) | -0.26 (-0.76, 0.23) | -0.05 (-0.67, 0.57) |  | 0.01 (-0.16, 0.19) | 0.07 (-0.12, 0.25) | -0.14 (-0.48, 0.20) |  | 0.10 (-0.55, 0.74) | 0.30 (-0.68, 1.29) | 0.30 (-1.25, 1.85) |
| quantile=0.25 | 0.12 (-0.18, 0.43) | -0.32 (-0.86, 0.23) | -0.44 (-0.98, 0.11) |  | 0.02 (-0.12, 0.15) | 0.00 (-0.26, 0.27) | 0.03 (-0.26, 0.33) |  | 0.05 (-0.63, 0.72) | 0.44 (-0.57, 1.44) | -0.52 (-2.68, 1.65) |
| quantile=0.50 | -0.04 (-0.47, 0.38) | -0.26 (-1.08, 0.56) | -0.75 (-1.51, 0.00) |  | 0.05 (-0.12, 0.22) | 0.14 (-0.20, 0.49) | -0.22 (-0.62, 0.17) |  | 0.08 (-0.98, 1.13) | 0.28 (-2.78, 3.33) | -1.75 (-5.59, 2.09) |
| quantile=0.75 | 0.12 (-0.50, 0.73) | 0.12 (-0.69, 0.93) | -0.80 (-2.12, 0.52) |  | 0.02 (-0.26, 0.31) | 0.22 (-0.32, 0.75) | -0.08 (-0.69, 0.53) |  | -0.96 (-3.48, 1.55) | 1.92 (-2.85, 6.69) | -1.10 (-8.36, 6.16) |
| quantile=0.90 | 0.03 (-1.11, 1.18) | 0.16 (-1.21, 1.53) | -0.58 (-1.61, 0.44) |  | -0.19 (-0.58, 0.19) | 0.22 (-0.58, 1.02) | 0.42 (-0.69, 1.54) |  | 0.22 (-4.75, 5.19) | 3.52 (-7.70, 14.73) | -8.32 (-29.89, 13.24) |
